# Quantum Capacitance of Two-Dimensional-Material-Based Supercapacitor Electrodes

**DOI:** 10.1021/acs.energyfuels.3c02714

**Published:** 2023-10-26

**Authors:** Subrata Ghosh, Sushant K. Behera, Ashutosh Mishra, Carlo S. Casari, Kostya Ken Ostrikov

**Affiliations:** † Micro and Nanostructured Materials Laboratory (NanoLab), Department of Energy, Politecnico de Milano, Via Ponzio 34/3, Milano 20133, Italy; ‡ Department of Materials Engineering, Indian Institute of Science, Bengaluru, Karnataka 560012, India; § Department of Applied Mechanics, Motilal Nehru National Institute of Technology Allahabad, Prayagraj, Uttar Pradesh 211004, India; ∥ School of Chemistry and Physics and QUT Centre for Materials Science, Queensland University of Technology (QUT), Brisbane, Queensland 4000, Australia

## Abstract

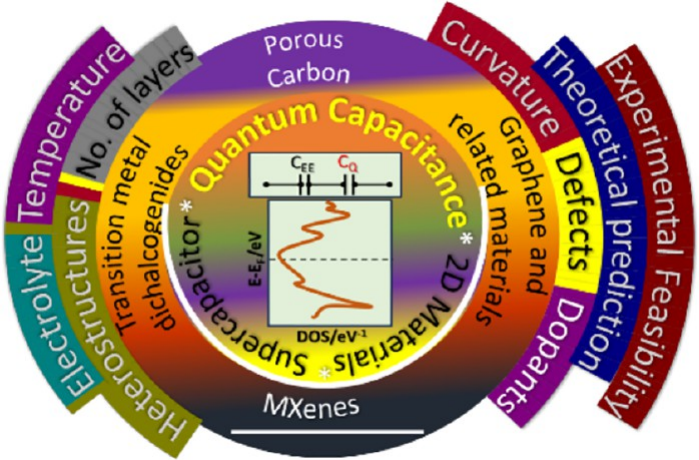

Electrochemical energy storage technology has emerged as one of the most viable solutions to tackle the challenge of fossil-fuel-based technology and associated global pollution. Supercapacitors are widely used for high-power applications, and there is tremendous ongoing effort to make them useful for high-energy storage applications. While electrode materials of supercapacitors play a central role in charge storage performance, insights into the contribution from different charge storage mechanisms are crucial from both fundamental and applied aspects. In this context, apart from the electric double layer and fast redox reaction at/near the surface, another pronounced contribution from the electrode is quantum capacitance (*C*_Q_). Here, the origin of *C*_Q_, how it contributes to the total capacitance, the possible strategies to improve it, and the state-of-art *C*_Q_ of electrode materials, including carbon, two-dimensional materials, and their composites, are discussed. Although most of the studies on quantifying *C*_Q_ are theoretical, some case studies on experimental measurements using standard electrochemical techniques are summarized. With an overview and critical analysis of theoretical studies on quantum capacitance of electrode materials, this review critically examines the supercapacitor design strategies, including choosing the right materials and electrolytes. These insights are also relevant to other types of clean energy storage technologies, including metal-ion capacitors and batteries.

## 1 Introduction

A supercapacitor (SC, also commonly termed as an electrochemical capacitor) is one of the rapidly emerging electrochemical energy storage devices for diverse clean energy technologies. Indeed, it can store a charge around 10–100 times higher than the conventional dielectric capacitor and is well-known for its superiority in high-power applications over conventional batteries.^1^ In terms of energy and power densities, a supercapacitor delivers a higher (lower) energy density than the conventional capacitor (battery) and has a higher power density and cycle life than the battery (Table 1).^2^ The reason behind the effective usage of SC in power applications is the excellent power density and prolonged life cycle. This makes SC a popular candidate in applications, such as hybrid electric vehicles, grid stabilization systems, forklifts, load cranes, aerospace equipment, etc. The global challenge of SCs is to enhance the energy density to compete with established battery technologies.

**Table 1:** Comparison Table of Supercapacitors, Conventional Capacitors, Metal-Ion Batteries, Metal-Ion Capacitors, and Redox Flow Batteries^3−6^

	supercapacitor	capacitor	Li-ion battery	Li-ion capacitor	redox flow batteries
storage mechanism	physical	physical	chemical	chemical and physical	chemical
operating voltage (V)	1.0–3.2	4–630	2.5–4.3	2.2–3.8	1.0–2.1
energy density (Wh/kg)	2.5–15	<0.1	75–250	10–100	<10–70
power density (W/kg)	500–10000	>1000000	150–315	300–5000	10–100
cycle life	>10^5^	>10^6^	10^3^–10^4^	10^4^–10^6^	>10^3^
operating temperature (°C)	from −40 to 70	from −20 to 100	from −20 to 60	from −25 to 85	from 0 to 60
efficiency (%)	95	99	85–90	90	60–85
self-discharge	very high	very high	very low	low	low
safety	good	good	needs improvement	good	low
voltage monitoring	not required	not required	needed	needed	

Like other electrochemical energy storage devices, SCs mainly consist of electrode materials and electrolytes. Thus, one can easily identify that the electrode materials are one of the keys to enhance the energy density of the device (*E*) via the relation *E* = 1/2*CV*^2^, where *C* is the specific capacitance and *V* is the voltage of the device. There are two types of energy storage mechanisms in SCs: one is storing the charge via double-layer formation at the electrode/electrolyte interface, and the other mechanism is based on the rapid redox reactions at the surface or near-surface. The former mechanism is known as the electric double-layer capacitance (EDLC), whereas the latter mechanism is called the pseudocapacitance. Among them, the pseudocapacitor can store 10–100 times higher charge than the electric double-layer (EDL) capacitor, whereas the latter features excellent electrochemical stability, rate performances, and better charge-transfer kinetics.

**Figure 1:**
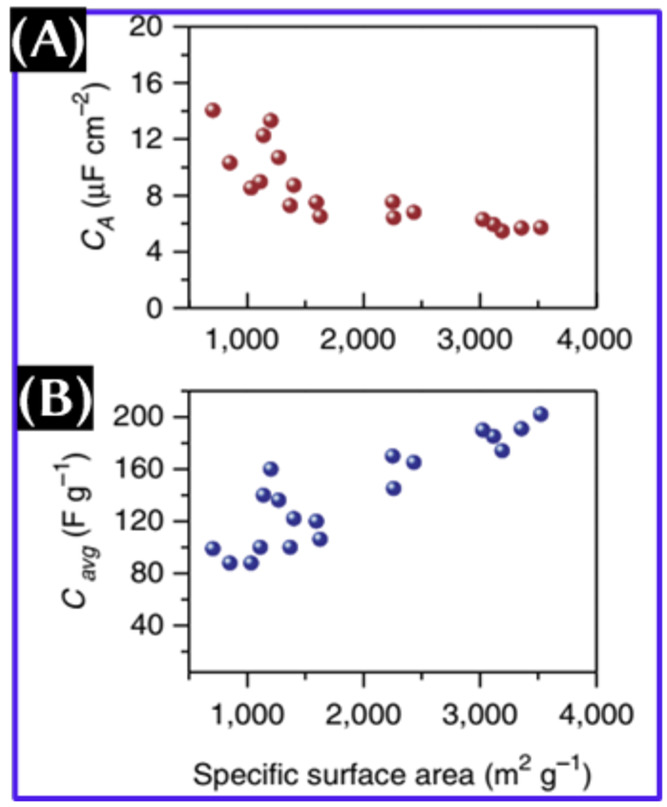
Areal capacitance (orange) and gravimetric capacitance (blue) of porous carbon with respect to the specific surface area. This figure was reproduced with permission from ref (7). Copyright 2014 Springer Nature, Limited.

In the case of the EDLC mechanism, the specific (or areal) capacitance (*C*_dl_) is related to the surface area (*A*) of active materials as
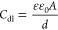
1where ε_0_ = 8.85 × 10^–12^ F/m, ε is the dielectric constant of the electrolyte, *A* is the surface area, and *d* is the radius of the counterions. It can be seen from Figure 1 that the gravimetric capacitance of porous carbon increases with the surface area, and unfortunately, the areal capacitance shows the opposite trend.^7^ Apart from the gravimetric capacitance, the high areal and volumetric capacitances make it highly desirable to use the SC for high-power applications over a small footprint area. Importantly, considering the surface area of 1500 m^2^/g, *C*_dl_ should be 30–70 μF/cm^2^ depending upon the choice of electrolyte. However, the areal capacitance obtained with porous carbon was only 4–5 μF/cm^2^.^7^ Furthermore, graphene, with a theoretical surface area of 2600 m^2^/g, showed the areal (gravimetric) capacitance of 13.5 μF/cm^2^ (355 F/g),^7^ and activated microwave-expanded graphite oxide with a surface area of 3100 m^2^/g exhibited the areal (gravimetric) capacitance of 6 μF/cm^2^ (200 F/g) in an aqueous electrolyte.^8^ Although predicted, the gravimetric capacitance of graphene of around 550 F/g has not been achieved thus far. These observations certainly ensured that increasing the surface area is not the only solution for enhancing the specific capacitance. Moreover, the higher surface area reduces the volumetric capacitance of the supercapacitor electrodes.

The lower values of capacitance and energy density of carbon-based materials are due to the low packing density, chemical inertness, hydrophobicity in aqueous electrolyte, and low density of states (DOS).^9,10^ To enhance the storage performance, the common strategies are doping or functionalizing,^9^ introducing defects,^11^ and designing heterostructures with pseudocapacitive material (metal-based materials, conducting polymers, etc.).^12^ In all cases, one can see that the major changes in the pristine structure are surface area modification and micro-, macro-, and mesopore optimization for the effective electrolyte ionic accessibility to the entire surface of an electrode, improvement in electrical conductivity and/or structure for better charge-transfer kinetics, changes in wettability to improve the electrode/electrolyte interaction, etc. To explore the phenomena at the surface in dynamic conditions (during charging/discharging), major attention is given to the *in situ* investigations by Raman spectroscopy, X-ray diffraction techniques, microscopic techniques, etc. These investigations provide a wealth of information to understand the charge storage mechanisms and help improve the electrode material structure, morphology, and property by means of standard synthesis and/or post-synthesis methods, to choose the appropriate electrolyte or the electrolyte modification, etc. Besides the changes in the structural, morphological, and other intrinsic properties of pristine materials by the above-mentioned methods, electronic DOS is also affected. While functional groups or dopants and heterostructures mostly contribute via pseudocapacitance, the changes in the DOS of electrode material are responsible to contribute to quantum capacitance (*C*_Q_). While modifying the carbon structures with the above-mentioned methods, one can notice that the modified structure can provide a higher surface charge density and higher *C*_Q_ in either positive or negative bias. This fact is quite important to decide whether the electrode can be used for a symmetric supercapacitor or an asymmetric supercapacitor.

Besides carbon-based materials, such as MXene, and transition metal chalcogenides also provide quantum capacitance that can be increased further by modifying the structure. The physical properties of carbon and two-dimensional (2D) material electrode are summarized in Table 2. However, modification does not always enhance the quantum capacitance of the final structures, and one needs to pay attention to the structural stability as well. Some other factors, like doping and defect concentration, number of layers, and curvatures, also have a significant impact on the quantum capacitance. It has also been seen from the simulation results that quantum capacitance also depends upon the type of electrolyte ions and the temperature.^13−17^ Hence, a proper combination of pristine electrode materials, heteroatoms, defects, and types of electrolyte ions is necessary to obtain the best quantum capacitance value and, hence, the total capacitance, energy/power density, and other key parameters of SCs.

**Table 2:** Comparison of Physical Properties of Carbon and 2D Materials

material	element and hybridization	surface area (m^2^/g)	electrical properties or electrical conductivity	normalized capacitance of the supercapacitor
porous carbon^7^	carbon with all hybridization	∼3500 (maximum)	10^–2^–10^3^ S/cm	4–5 μF/cm^2^
graphene	sp^2^-bonded carbon	2600 (theoretical)	∼10^8^ S/cm	13.5 μF/cm^2^ (355 F/g);^7^ 300 F/cm^3^ ^18^
graphyne	sp–sp^2^-bonded carbon	5510 (theoretical)^19^		
carbyne	sp carbon	13000 for H_2_	tunable depending upon the bonding	
transition metal dichalcogenides	MX_2_, a layer of transition metals (Mo, W, Ta, etc.) sandwiched between two layers of chalcogens (S, Se, and Te)	up to ∼200 depending upon morphology	semimetal or semiconducting	400–700 F/cm^3^ ^20^
MXene	M_*n*+1_X_*n*_T_*x*_; 2D metal carbide/nitride, where M is a metal, X is C and N, and T is a functional group (e.g., O, F, OH, and Cl)	up to around 200 depending upon morphology^21^	metallic or semiconducting	1500 F/cm^3^ or 380 F/g^22^

## 2 Scope and Structure

**Figure 2:**
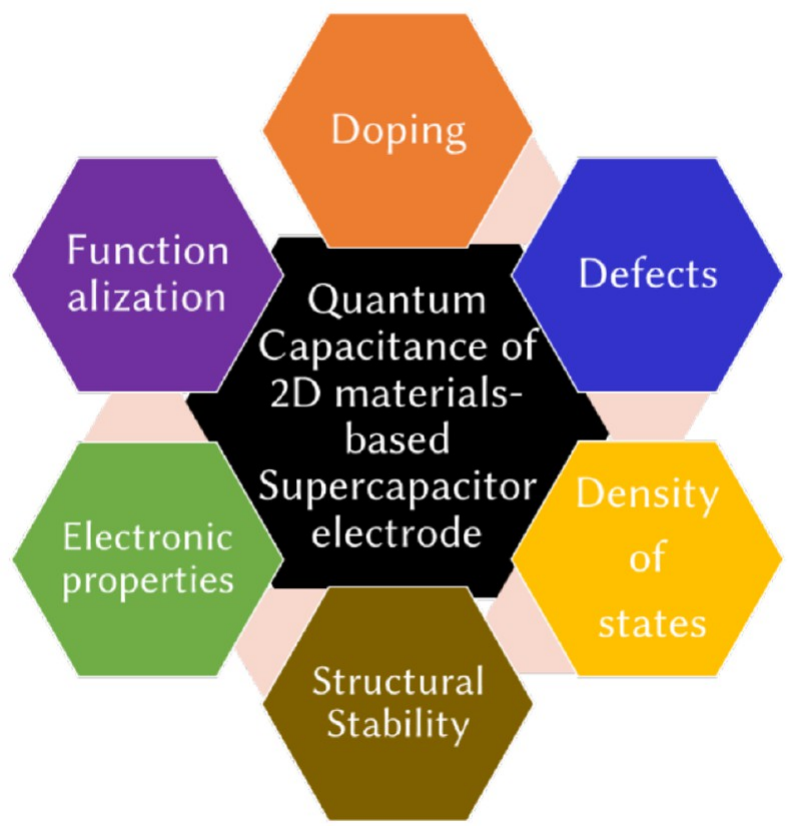
Scope and structure of this review.

**Figure 3:**
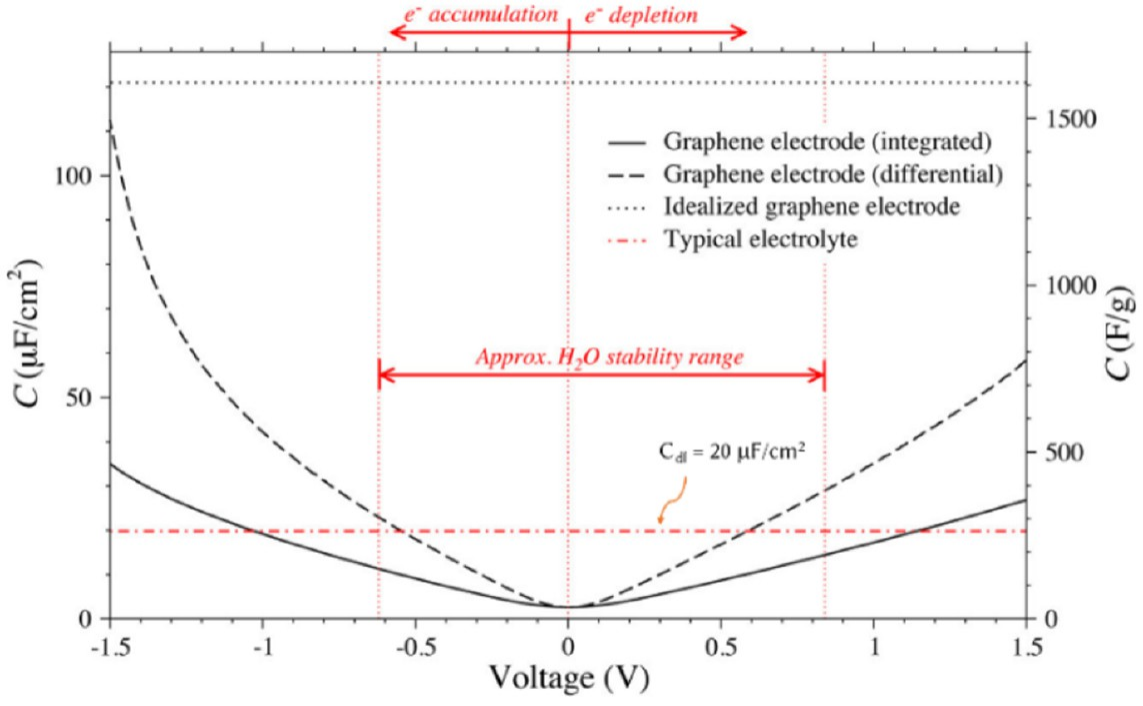
Integrated (solid black line) and differential (dashed black line) quantum capacitances and double-layer capacitance (red dashed line) for pristine graphene. The calculation is based on the fixed-band approximation. Aqueous electrolytes can be operated within the H_2_O stability range, whereas a wider window is used for organic/ionic electrolytes. This figure was reproduced with permission from ref (24). Copyright 2013 American Chemical Society.

There is plenty of theoretical research based on density functional theory (DFT), first-principles calculations, etc. to understand the importance of *C*_Q_ and how it can be tailored. Indeed, there are some experimental attempts to evaluate *C*_Q_ using the standard electrochemical characterization approach. However, there is no exhaustive coverage on this specific topic of quantum capacitance of supercapacitor electrodes, except the partial discussion in ref (23). This review is intended to serve as a supplement to the many comprehensive theoretical results and discussions of the most recent results dealing with the quantum capacitance of the supercapacitor electrode (Figure 2). To provide insights and updates on this topic, the key considerations are summarized as follows:

- This review is based on the theoretical or simulation results published in the cited articles. Thus, we are encouraging authors to read the cited references for the detailed simulation methods. Indeed, we summarized the outcome of the theoretical results with critical analysis and discussed the experimental feasibility of the proposed results based on the simulations.
- On the basis of the literature review, the cited theoretical article reports either the integrated quantum capacitance or differential quantum capacitance based on the method used. For example, the integrated capacitance is derived for continuum capacitance modeling, and differential capacitance is for first-principal calculations.^24^ Experimentally, integrated or differential capacitance can be obtained from cyclic voltammetry and galvanostatic measurements, whereas differential capacitance can be obtained from impedance spectroscopy. There is certainly a difference between the value obtained for the integrated and differential *C*_Q_ of the electrode, as shown in Figure 3.^24^ For the sake of simplicity, we mentioned the maximum *C*_Q_ of electrode materials obtained using the theoretical simulations for all references cited.
- In all reports, DOS calculation has been carried in the range from −0.6 to +0.6 V for aqueous electrolytes and from (≤)–1.2 to (≥)1.2 V for ionic/organic electrolytes (Figure 3). This fact can be identified from the potential window (V) used in the *C*_Q_–*V* plot.

In the present review, section 3 provides a detailed conceptual background of quantum capacitance. Sections 4–7 discuss the quantum capacitance of various electrode materials based on carbon, 2D materials, and their heterostructures and the possible strategies to enhance it further. The 2D-material-based supercapacitor electrodes are under investigation as a result of their high surface area, rapid charge/discharge capabilities, excellent electrical conductivity, tailorable properties, and potential for flexible and environmentally sustainable energy storage solutions, driving innovation in energy storage technologies. In sections 8 and 9, one can see the dependency of *C*_Q_ upon the temperature and electrolyte used, respectively. While discussing the quantum capacitance of the supercapacitor electrode materials, we also shed light on the experimental viewpoints or the experimental feasibility of the theoretically predicted strategies to enhance *C*_Q_. Section 10 deals with the experimental approach to measure *C*_Q_ of the electrode using standard electrochemical characterization. Finally, the challenges and outlook are discussed in section 11.

## 3 Quantum Capacitance (*C*_Q_)

**Figure 4:**
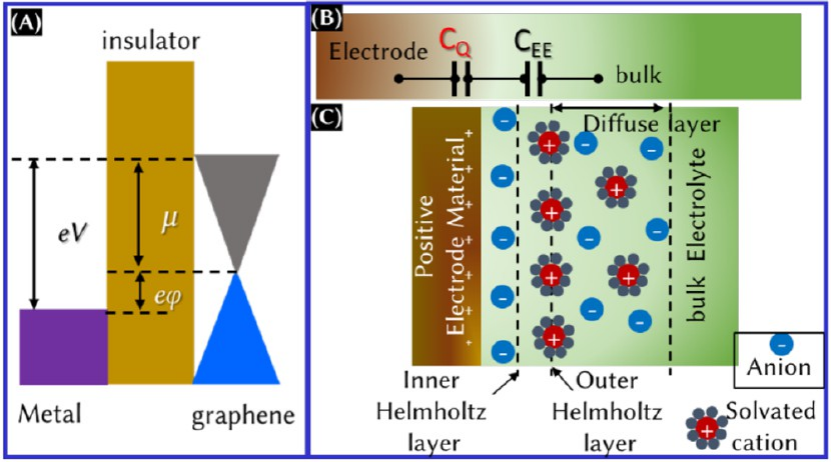
(A) Schematics of metal–insulator–graphene capacitors with a band diagram at a finite bias, (B) schematic of the equivalent circuit with the double-layer capacitance (*C*_EE_) and *C*_Q_, and (C) electric double-layer formation model.

To explore the concept of quantum capacitance, let us consider the parallel-plate capacitor consisting of 2D metal (graphene, in this case) and normal metal electrodes separated by insulating materials, as shown in Figure 4A.^25^ The difference in electrochemical potential (V) can be expressed as *eV* = *e*φ + μ, where φ is the potential drop between the electrodes and μ is the chemical potential. Upon differentiation of the above equation with respect to the carrier concentration
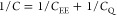
2and
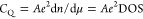
3where *C*_EE_ is the capacitance as a result of the electrode/electrolyte interaction, *n* is the carrier concentration, and d*n*/dμ is the thermodynamic DOS (Figure 4B). The contribution of *C*_Q_ for metal electrodes is negligible, because they have infinite DOS near the Fermi energy level. However, the contribution is significant for the electrode materials only when the magnitudes of *C*_dl_ (for Figure 4B, *C*_EE_ = *C*_dl_) and *C*_Q_ are in a similar order. Because the electrochemical energy storage device is a combination of electrodes and electrolytes (panels B and C of Figure 4), one can easily validate eq 1 that the total capacitance is the combination of capacitance as a result of the electrode/electrolyte interactions (double-layer capacitance and/or pseudocapacitance) and the intrinsic capacitance of the electrode (quantum capacitance). The double-layer capacitance is in series with quantum capacitance, as seen in Figure 4B.

*C*_Q_ is an intrinsic property of the materials, which arises from the kinetic, exchange–correlation, and electron–phonon interaction energies in the total energy functional.^26,27^ For the graphene–vacuum–graphene capacitor, considering the kinetic term *k*_B_*T* ≪ *eV*, *C*_Q_ can be expressed as^24^
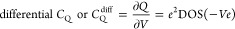
4or because the total energy storage capacity is based on the integrated value over a complete charge–discharge cycle, integral *C*_Q_ can be expressed as
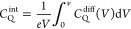
5Thus, one can define the capacitance associated with the DOS as the quantum capacitance. Therefore, increasing the density of states and, hence, quantum capacitance is another solution to increase the total capacitance. It is important to note that *C*_Q_ cannot be zero at room temperature and has a minimum positive value at 0 V because there is always thermal broadening of the electron energy distribution.^28^

## 4 Carbon-Based Materials

Among the SC electrode materials, the mostly studied and even commercially used materials (e.g., activated carbon) are carbon-based materials.^29−31^ The reason behind this is, as the activated carbon stores the charge via a EDLC (a physical mechanism) and, hence, better power density, cycle life, and chemical stability can be obtained, surface morphology and porosity can be tailored. Carbon-based materials offer certain benefits, such as having different dimensionality [from zero-dimensional (0D) to three-dimensional (3D)], and their optical, electrical, mechanical, biocompatible, and other properties can be effectively controlled.^9,32^ The main carbon nanostructures discussed in this section are graphene, carbon nanotubes (CNTs), graphyne, etc. Thanks to the unique properties and structure of graphene that are quantified by numerical modeling and simulations, graphene engineering becomes possible. Thus, graphene is adapted as a model structure. Moreover, by rolling, folding, cutting, etc., one can transform graphene into carbon nanotubes, fullerene, and graphene nanoribbons. If CNTs are taken as an example, strain, curvature, and chirality (armchair or zigzag, which define its conducting properties, like metallic or semiconductor) should be considered. Importantly, reduced graphene oxide (RGO) or graphene nanoplatelets (GNPs) cannot be defined as graphene; they are the members of the wider graphene family. Unfortunately, RGO or GNPs are termed as graphene in many works, while we are strict on the definition of graphene, where sp^2^-bonded carbon is arranged in a single-layer hexagonal honeycomb lattice and without attached functional groups. Because the *C*_Q_ value of pristine graphene is limited, it is possible to increase it by adjusting the number of graphene layers,^7^ doping or functionalizing,^33^ surface rippling, and causing tensile strain,^24^ as discussed below.

### 4.1 Effects of the Number of Graphene Layers and Local Strain

**Figure 5:**
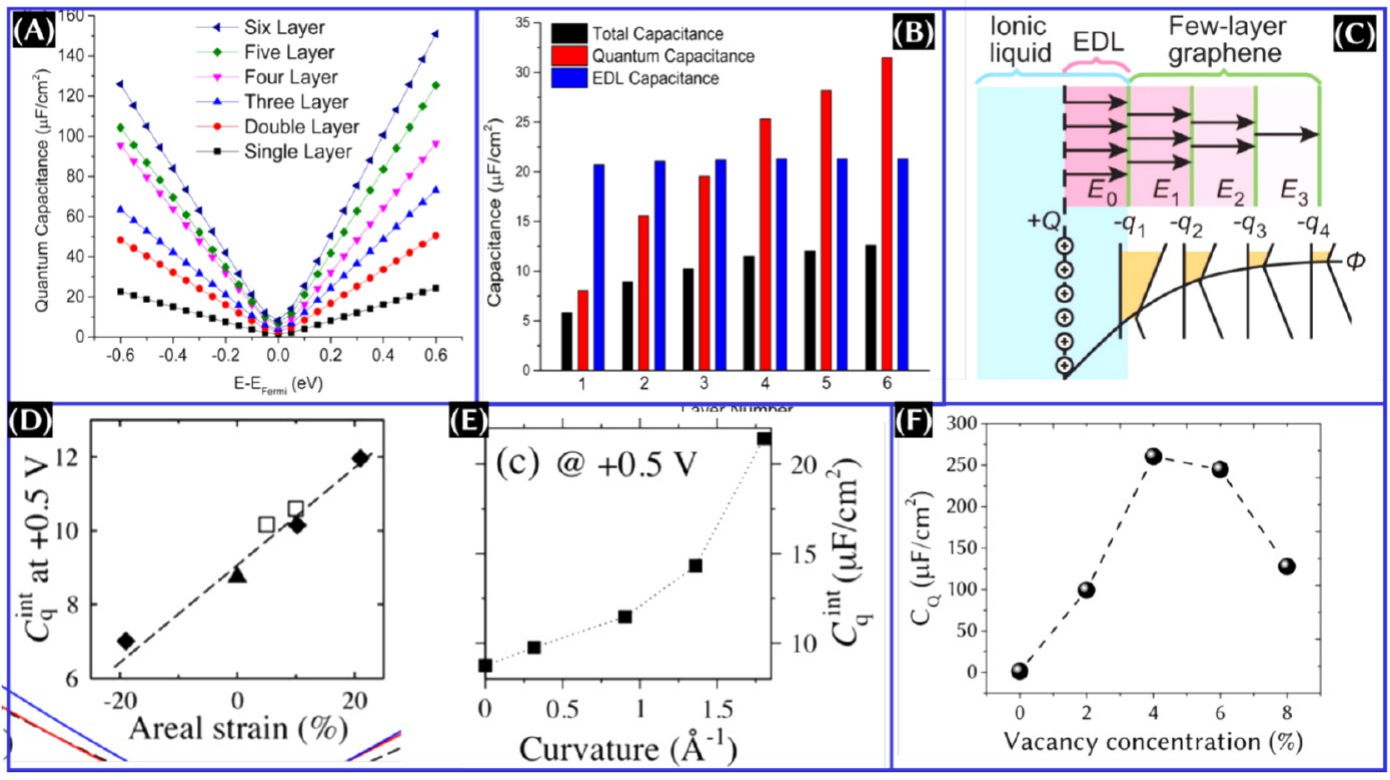
(A) Quantum capacitance of graphene with respect to the number of layers in a 1.0 M NaF aqueous electrolyte and (B) *C*_Q_, *C*_dl_, and total areal capacitance of graphene with respect to the number of layers. These panels were reproduced with permission from ref (34). Copyright 2015 American Chemical Society. (C) Schematic of the charge distribution of few-layer graphene in ionic liquid showing the screening effect. This panel was adapted with permission from ref (36). Copyright 2013 Nature Portfolio. Integrated *C*_Q_ of graphene at different applied (D) axial strains and (E) curvature. These panels were reproduced with permission from ref (24). Copyright 2013 American Chemical Society. (F) *C*_Q_ of graphene with respect to the vacancy defect concentration. Data for panel F are taken from the text of ref (13).

Dependent upon the number of layers, one can categorize them into graphene (one layer only), bilayer graphene, trilayer graphene, few-layer graphene, or multi-layered graphene (number of layers being ≥4). It is well-known that the intrinsic properties of graphene strongly depend upon the number of layers. It is quite interesting, as seen from Figure 5A, that the DOS and, hence, *C*_Q_ can be enhanced by increasing the number of layers, and one can reach the saturation value (mostly after four layers), beyond which the enhancement in *C*_Q_ is quite negligible. On the basis of the theoretical results, the effect of the graphene layer on *C*_Q_ can be realized (Figure 5B), and one needs to pay attention that the number of layers of graphene has an insignificant impact on *C*_dl_.^34^ While increasing the number of layers, one also needs to consider the screening effect (Figure 5C). As a result, there will be space charge capacitance in series with *C*_dl_ and *C*_Q_. When another foreign atom is introduced in the parent matrix, the simulated maximum *C*_Q_ for N/P/Ti-doped multi-layered graphene is found to be 158.94 μF/cm^2^ at 0.54 V, which is even higher than that of pristine multi-layered graphene (3.55 μF/cm^2^ at 0 V). This result reflects that dopants or foreign atoms have a significant effect on the enhancement of *C*_Q_. More details are given in sections 4.3 and 4.4. However, it has also been predicted theoretically that co-doped multilayered graphene has a lower *C*_Q_ compared to co-doped single-layer graphene (163.19 μF/cm^2^ at positive bias), which is attributed to the interaction of dopants located in the adjacent layers.^35^ This observation suggests that merely increasing the number of graphene layers of the doped graphene-based structures may not be the most effective way to boost DOS or *C*_Q_.

### 4.2 Strain, Curvature, and Defects in Graphene

Apart from the number of layers, the introduction of local strain (uniaxial or biaxial) and local curvature was also found to be influential in enhancing the quantum capacitance (panels D–E of Figure 5). Curvature, folding, or ripples are quite common in graphene when it is transferred to the desired substrate by standard transfer techniques, and they are common in graphene oxide when prepared, for example, by the Hummers method. Whatever the case, by increasing the number of layers or introducing strains or curvatures, the enhancement of *C*_Q_ may not be significant, as seen from panels A and B of Figure 5. Alternatively, the introduction of defects in the structure is promising and more feasible. Integrated *C*_Q_ for pristine graphene and graphene with a single vacancy (SV), double vacancy (DV), triple vacancy (TV), and quadruple vacancy (QV) was estimated, using DFT calculations, to be 10.72 μF/cm^2^ at 0.6 V, 107.61 μF/cm^2^ at −0.04 V, 39.16 μF/cm^2^ at 0.6 V, 160.29 μF/cm^2^ at 0.02 V, and 119.6 μF/cm^2^ at −0.06 V, respectively.^37^ This result reflects that a triple vacancy in graphene leads to the highest *C*_Q_. The defect concentrations for graphene with SV, DV, TV, and QV were 3.12, 6.25, 9.37, and 12.5%, respectively. Eventually, the vacancy defect was found to be more influential (120.72 μF/cm^2^) compared to the other types of defects, like the Stone–Wales defect (44.38 μF/cm^2^), where theoretically estimated *C*_Q_ of pristine graphene was 21.37 μF/cm^2^.^38^ It is important to note that modified graphene does not lose its conductivity even after introducing vacancy and Stone–Wales defects.^38^ The vacancy defect induces p-type behavior as a result of the electron deficiency and, hence, shifts the Fermi level into the valence band. However, there is a trade-off between *C*_Q_ and thermodynamic stability of defected graphene,^37^ which has been tackled by introducing nitrogen in the graphene matrix. The highest integrated *C*_Q_ (260.68 μF/cm^2^ at 0.04 V) with the least formation energy (0.25 eV/Å) was found from simulation results for single-vacancy trimerized nitrogen^37^ to lower the formation energy feasible to synthesize the material experimentally. It can be enhanced further by increasing the defect concentration up to a certain limit (Figure 5F).^13^

If we consider graphene-based materials synthesized at lab- or mass-scale, like graphene foam or vertical graphene nanosheets, for example, this 3D graphene structure unavoidably contains plenty of folded edges, with a local strain developed during the growth, vacancy, and boundary-like defects, and they are the structure with few layers of graphene (specifically, one can find single layer, bilayer, and/or few layers from the graphene-based structure). Indeed, the theoretical study reveals that the presence of edges on the graphene surface is also beneficial to improve the DOS.^39^ In particular, graphene nanoribbons with zigzag edges were found to have significant enhancement in *C*_Q_, and it increases further with the decrease of the nanoribbon width.^39^ Thus, one can say that a 3D structure consisting of few-layer graphene could be preferential over its single-layer counterpart, keeping the screening effect in mind. Furthermore, 3D graphene structures also provide plenty of electrochemically active surfaces compared to graphene, where only the top surface interacts with the electrolyte. Practically, both the higher areal and volumetric capacitances of vertical graphene nanosheets have been reported over the planar nanographitic structure.^40^ In this scenario, it is important to understand how the number of layers of graphene with curvatures and strain can be combined with doping or adsorbing foreign atoms to enhance the performance further.

### 4.3 Functionalization of Graphene

**Figure 6:**
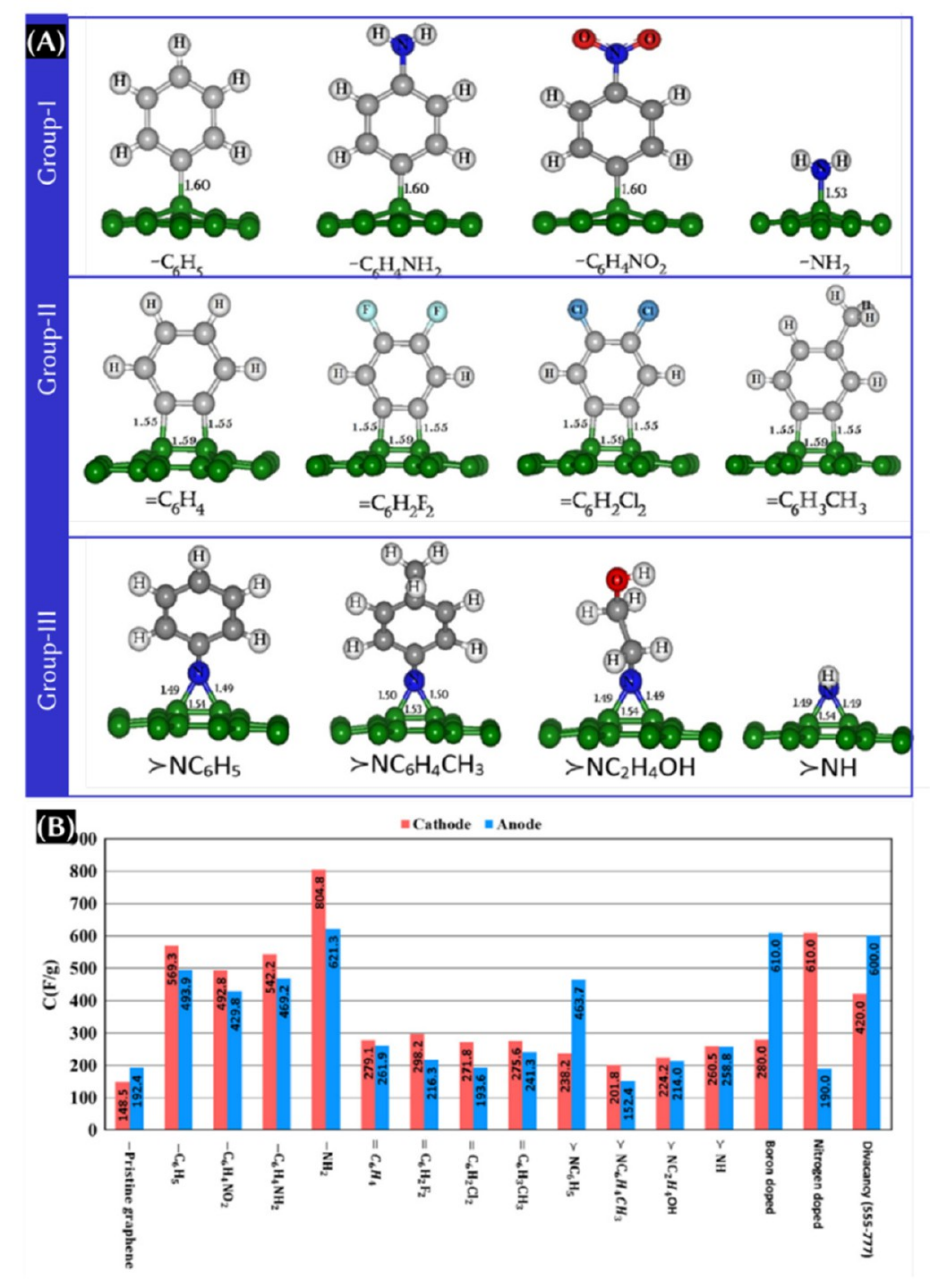
(A) Categories of substituted groups for functionalized grapheneL groups I, II, and III. (B) Theoretical integrated *C*_Q_ at the anode and cathode within the H_2_O stability voltage range for different functionalized graphene. This figure was reproduced with permission from ref (42) Copyright 2015 Elsevier, Ltd.

Graphene can be functionalized with various non-covalent aliphatic and aromatic molecules and radicals. The aliphatic group fragments include alkene, alkyne, ketones, amines, amides, nitriles, carboxylic acids, and sulfoxides. The aromatic molecules are benzene, aniline, phenol, anthracene, toluene, and naphthalene.^41^ The highest *C*_Q_ values obtained for the graphene functionalized with the acetone radical and phenol radical are 235 and 237 μF/cm^2^, respectively.^41^ The substituted group is categorized into groups I, II, and III, which is shown in Figure 6A.^42^ On the basis of the result obtained from this study,^42^ group I functionalized material can be used as both positive and negative electrodes, whereas group II and III functionalization is recommended as a negative electrode. Whatever the case, a recent study^42^ reveals that −NH_2_-functionalized graphene showed the highest theoretical integrated *C*_Q_ (Figure 6B).

**Figure 7:**
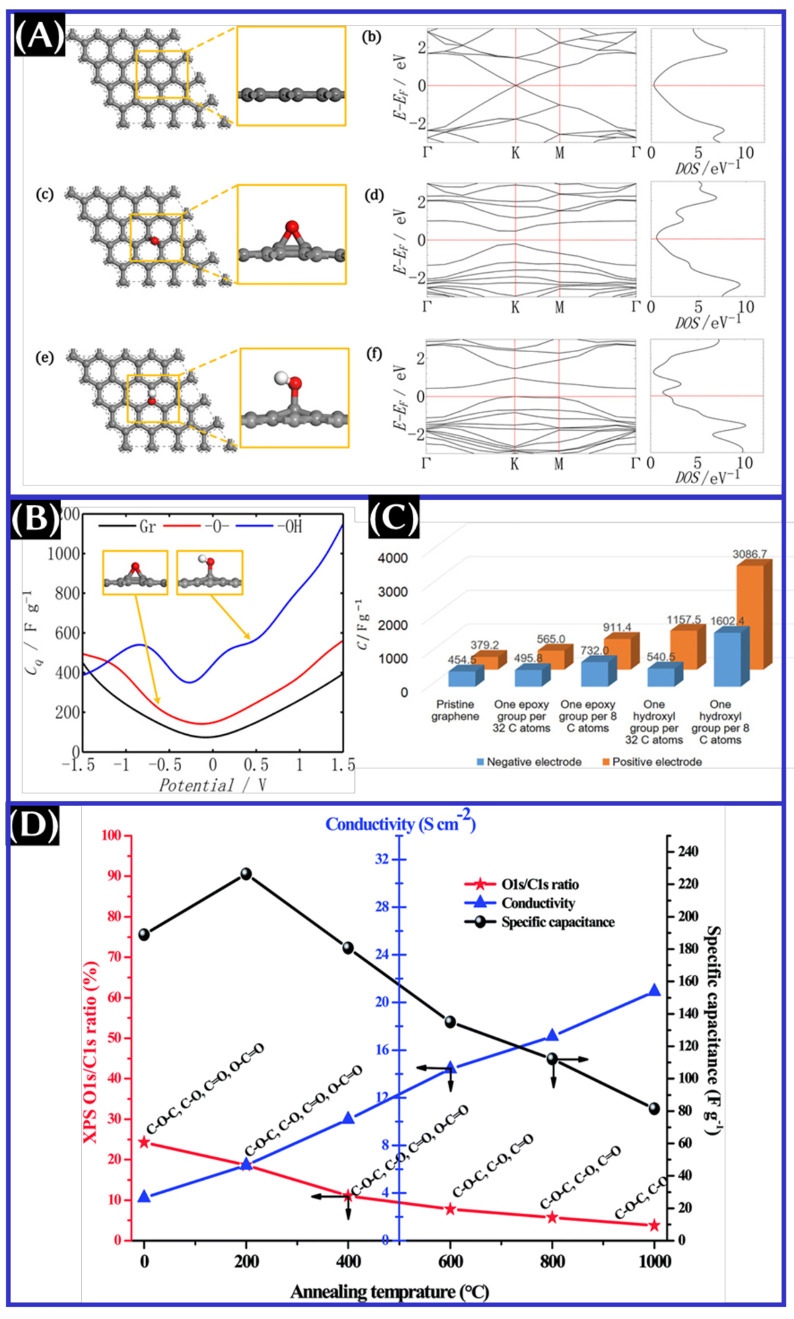
(A) DFT-optimized structures with corresponding band structures and DOS, (B) plot of quantum capacitance, and (C) capacitance at positive and negative biases of pristine graphene, graphene oxide with an epoxy group, and graphene oxide with a hydroxyl group. These panels were reproduced with permission from ref (49). Copyright 2018 Wiley-VCH Verlag GmbH & Co. KGaA. (D) Plot of physicochemical properties of graphene oxide versus the annealing temperature. This panel was reproduced with permission from ref (50) Copyright 2014 Royal Society of Chemistry.

The carbon surface, including most of the nanocarbons, is basically functionalized with physisorbed oxygen-related functional groups. This can be easily identified from the typical X-ray photoelectron spectra.^43^ However, the bare carbon surface is mostly hydrophobic in nature,^10^ except for highly porous amorphous carbon nanofoam prepared by pulsed laser deposition, for example, and this negligible amount of functional group may not have a significant impact on the charge storage contribution. Oxidizing the carbon surfaces enhances the number of electrons transferred to the sp^2^ network and also makes the surface near-hydrophilic or hydrophilic depending upon how the surfaces are activated (chemical activation, plasma functionalization, etc.).^44,45^ According to the DFT calculations, the electrochemically oxidized CNT yarn resulted in 0.016 electron transfer (0.001 for pristine CNT yarn) during the interaction with a single water molecule.^46^ On the other hand, graphene oxide (GO), which can be treated as highly oxidized graphene, has received significant attention as a result of its mass production, easy synthesis, low cost, and unavoidably relatively higher amounts of oxygen functional groups.^47,48^ The oxygen functional groups that are attached on the edge plane of GO in large quantities are epoxy and hydroxyl, whereas small amounts of carboxyl (−COOH) and carbonyl (C=O) are attached on the basal plane. Among them, the higher hydroxyl group (−C–OH) in the carbon matrix leads to a higher *C*_Q_, as obtained from the simulation result, which is attributed to the enhanced electronic states near the Fermi level (Figure 7A).^49^ Moreover, we emphasize that the −COOH groups on the graphene surface are found to be unstable experimentally with time, and an increase in the −COOH group resulted in lowered total geometric capacitance.^45^ Despite higher gravimetric capacitance obtained experimentally, GO showed degraded performance at a higher discharge rate.^50^ However, the transportation of electrons through the electrodes could be effectively enhanced by removing the functional groups from the surface. In other words, RGO provides better charge-transfer kinetics and conducting pathways for the electrons, but one needs to compromise the total gravimetric capacitance.^50^ Because RGO has very limited epoxy and hydroxyl groups, *C*_Q_ is found to be lower than that of GO (panels B and C of Figure 7).^49^ However, in comparison to the GO, graphene nanoribbons, anodic and cathodic electrochemically exfoliated graphene, and liquid-phase exfoliated graphene (95% capacitance retention after 15 000 cycles in most of these cases), it has been seen from the experimental investigation that RGO has poor cyclability (70%) and higher equivalent series resistance after several charge–discharge cycles.^47^ To obtain an overall idea of the differences in properties and performance between GO and RGO, see Figure 7D.

### 4.4 Doped Carbon

For oxygen, two configurations can be considered: oxygen functionalization (covered in section 4.3) and in-plane oxygen incorporation in the graphene matrix. The latter case can only be considered as oxygen doping. Indeed, the dopants that received significant attention to tailor the fundamental and charge storage properties of carbons are boron (B), nitrogen (N), phosphorus (P), sulfur (S), halogens (Cl, Br, and F), silicon (Si), etc., with each of them having their advantages and limitations. Hence, we refer to ref (9) for more detailed information on each doped graphene electrode performance. The major advantageous feature of doping includes that it does not add any additional mass and enhances the double-layer capacitances while improving the charge-transfer kinetics.^9^ Because one can tailor the doping concentrations, the number of dopants (co-doping), and the defect level associated with doping, there are multiple options to enhance the quantum capacitance of the electrode in the structures,^51−53^ which is discussed in the following sections.

How doping can enhance the *C*_Q_ value of the final structure can be followed from
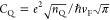
6where *n*_Q_ is the dopant-induced carrier density and *v*_F_ is the Fermi velocity. As mentioned above, dopants help to improve the stability of the structure while also changing DOS and, hence, *C*_Q_.

#### 4.4.1 Dopant Type

**Figure 8:**
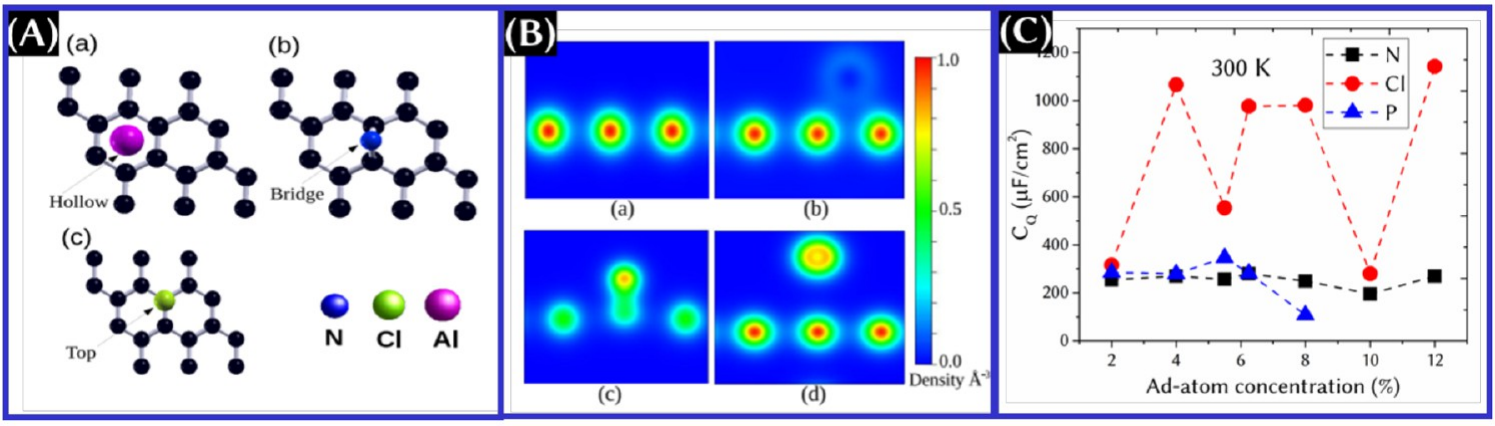
(A) Preferred adsorption position of dopants in the graphene matrix. (B) Contour plots for the electron density of pristine and doped graphene. These panels were reproduced with permission from ref (13). Copyright 2019 IOP Publishing, Ltd. (C) *C*_Q_ of doped graphene at different doping concentrations. To plot this, data are taken from the text of ref (13).

Obviously, each dopant has preferential adsorption sites on the graphene matrix (Figure 8A).^13^ One can also see the electron density associated with pristine and doped graphene in Figure 8B.^13^ Among the dopants, nitrogen is anticipated as the most promising and became more popular as a result of the (i) comparable size and ability to form strong valence bonds with carbon atoms, (ii) N doping being quite simple to manipulate the local electronic structures, and (iii) N–C bond distance being similar to the C–C bond length and, hence, the graphene symmetry being preserved even after N doping.^54^ Eventually, on the basis of the binding energy calculations, N-doped graphene is more stable than defected graphene.^55^ However, although the charge transfer/atom to N from the graphene sheet is 0.5e compared to that of P (0.3e),^54^ DFT calculations showed that P-doped graphene delivered higher *C*_Q_ (143.42 μF/cm^2^ at 0.57 V) than S-doped graphene (95.78 μF/cm^2^ at 0.54 V) and N-doped graphene (135.50 μF/cm^2^ at 0.23 V).^35^ Moreover, P-doped graphene has new states around the Fermi level without any shift^56^ and showed the capability to store the charge at an extended potential window in an aqueous electrolyte.^57^ On the basis of theoretical studies, Cl doping allows for the highest *C*_Q_ values to be obtained in comparison to doping with other elements, as evidenced by the maximum charge redistribution on graphene, shown in Figure 8B. This result reveals that the charge redistribution of doped graphene is another key parameter to obtain higher *C*_Q_. Importantly, one must pay attention to the atomic radius of the dopant. The dopant with a larger size than the carbon atom can produce severe stress in the structure, and hence, there will be failure of structural stability of the doped structure.

#### 4.4.2 Doping Concentration

Let us look at the effect of the doping concentration on *C*_Q_ of the final structure. At higher doping concentrations, *C*_Q_ is found theoretically to be higher for N-doped graphene (Figure 8C) and the structure is found to be more stable.^58^ However, there should be an optimum level of doping concentrations, and it may not have a monotonic relationship (Figure 8C). Beyond this, the doped structure can be unstable, and *C*_Q_ was found to be reduced.^13,58^ Moreover, at a higher Cl-doping concentration, a stronger interaction of Cl–Cl can take place, leading to the Cl_2_ formation with a possibility of desorption from the surface. Noteworthy, the interactions between the layers could also lower *C*_Q_ compared to the doping on single-layer graphene, except the Al case among B, N, and P doping.^54^

#### 4.4.3 Doping Configuration

Many configurations exist for the dopants on the parent matrix. Considering nitrogen as a dopant for graphene, the possible configurations are pyridine (N-6), pyrrolic N/pyridone N (N-5), quaternary/graphitic nitrogen (N-Q), oxidized pyridine N (N-X), and nitrogen oxide (N-Ox). It has been predicted that *C*_Q_ increases with N-6 and N-Q concentrations and remains constant with the pyrrolic N concentration.^59^ Pyrrolic N has a very small contribution to the total capacitance compared to pristine graphene. For the pyrrolic N case, there is an extra electron from the N atom in the p_*z*_ orbital because the N–H bond formation is balanced by the loss of one electron in the delocalized π bond as a result of the associated C vacancy. Among the possible configurations, N-6 shows higher *C*_Q_ than N-Q and pyrrolic N.^59^ On the contrary, the maximum *C*_Q_ follows the descending order as pyrrolic (195.12 μF/cm^2^ at −0.18 V) > graphitic (123.77 μF/cm^2^ at −0.54 V) > Stone–Wales (110.85 μF/cm^2^ at −0.41 V) > pyridinic (91.39 μF/cm^2^ at 0.6 V) > pristine graphene (22.17 μF/cm^2^ at −0.6 V).^51,60^ Surprisingly, the maximum *C*_Q_ is found to be increased theoretically to 215.55 μF/cm^2^ at −0.07 V when combined with Stone–Wales defects and 486.32 μF/cm^2^ at 0.2 V at 6.38% concentration of pyrrolic N.^60^ When the stability of the structure is considered, the graphitic structure stands out as a better candidate compared to the pyridinic and pyrrolic structures being the least stable structures.^60^ Thus, the discrepancy between the above reports on the role of pyrrolic N needs further clarification.

**Figure 9:**
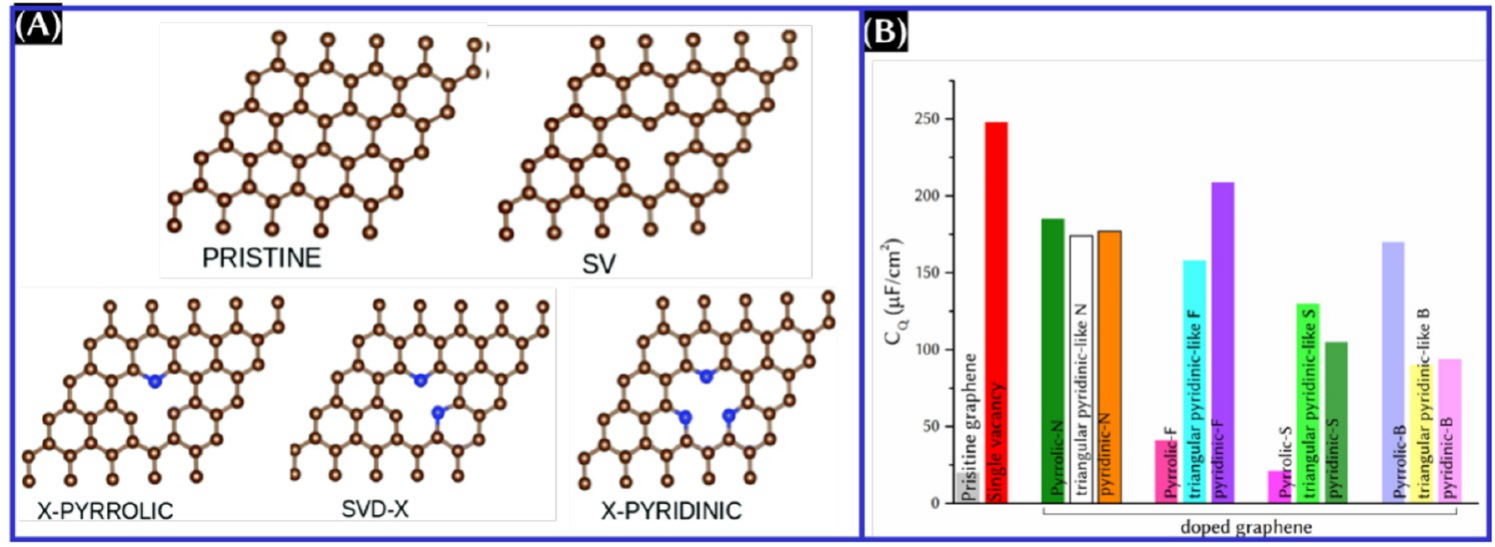
(A) Atomic presentation and (B) maximum *C*_Q_ of doped graphene with different doping configurations. Panel A was reproduced with permission from ref (51). Copyright 2020 Royal Society of Chemistry. For the plot of panel B, data are taken from the text of ref (51).

Figure 9 summarizes the simulation result of the dopant configuration-dependent *C*_Q_ of doped graphene. Three different configurations for dopants (X = N, F, S, and B) taken into consideration are pyrrolic X, triangular pyridinic-like X doped (SVD-X), and pyridinic X (Figure 9A).^51^ From the theoretical comparison, the *C*_Q_ value is the highest for a single-vacancy (SV)-defected graphene compared to doped graphene. Among the doping configurations, pyrrolic N and pyrrolic B exhibited the highest *C*_Q_, whereas the highest maximum *C*_Q_ is obtained from pyridinic F and SVD-S (Figure 9B). However, more systematic investigations on the configuration dependent upon dopants, such as P, Cl, and Si, on the quantum capacitance is not investigated theoretically thus far to the best of our knowledge via the literature survey and could be a subject of future research.

#### 4.4.4 Co-doping

**Figure 10:**
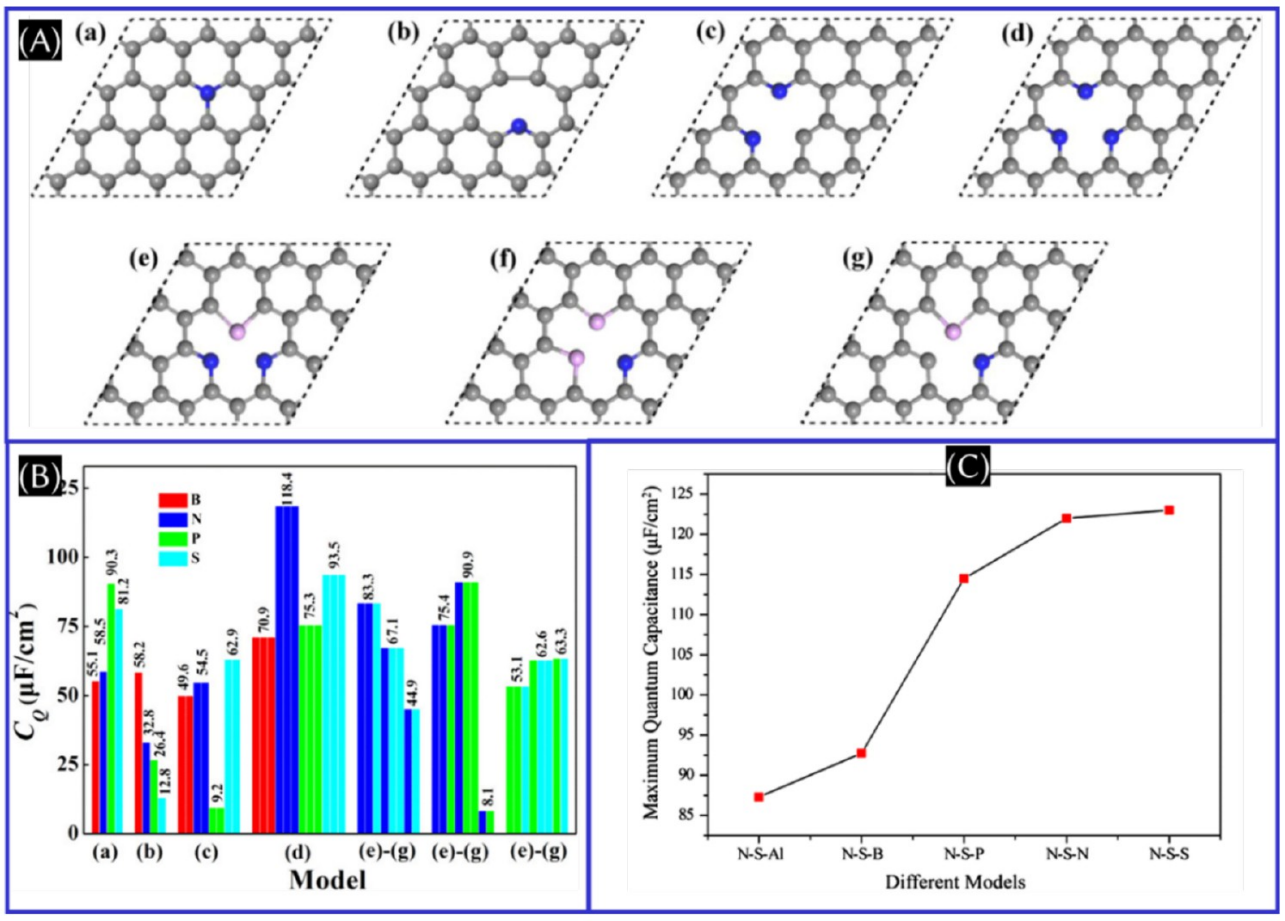
(A) Proposed doping model of graphene: (a) quaternary N (B, P, and S)-doped graphene (model a), (b–d) single-vacancy graphene with the single pyridine N (B, P, and S) doping (model b), double N (B, P, and S) doping (model c), and triple N (B, P, and S) doping (model d), and (e–g) single-vacancy graphene with NNS(P) co-doping (model e), NSS(P) co-doping (model f), and NS(P) co-doping (model g). (B) corresponding *C*_Q_ of doped graphene. These panels were adapted with permission from ref (58). Copyright 2019 American Chemical Society. (C) Plot of the maximum quantum capacitance of co-doped graphene. This panel was reproduced with permission from ref (62). Copyright 2019 Springer Nature.

Co-doping is found to be an appealing approach because it provides more stability over single doping with vacancies^58^ and also generated new electronic states near the Fermi level, and as a consequence, there is a significant enhancement in charge accumulation and *C*_Q_.^61^ To be specific from the DOS calculations, the new localized states have been seen near Fermi level DOS for pyridine B-doping among single doping with model b [single-vacancy graphene with single pyridine N (B, P, and S) doping], for all doping with model c [single-vacancy graphene with the double N (B, P, and S) doping], except P doping, which has no localized states near the Fermi level, and all doping for the triple B, N, P, and S doping with model d (Figure 10A).^58^ Among the chain model structures (N–N–N, N–N–S, N–S–N, N–S–S, S–N–S, and S–S–S), N–S–S is found theoretically to be the most stable structure and provides the highest *C*_Q_, which ensures that the 1:2 ratio of N/S co-doping is the most optimum ratio (panels B and C of Figure 10).^62^ On the contrary, the DFT calculation suggests that a low S concentration is preferable to achieve the highest *C*_Q_ for N/S co-doped graphene.^55^ It has also been reported that the triple N doping with a single vacancy [model d: single-vacancy graphene with the triple N (B, P, and S) doping] performed best at a positive potential and triple S doping with a single vacancy performed best at a negative potential.^58^ One needs to note that the co-dopants occupying the sites near the vacancy are quite stable compared to the occupied sites away from the vacancy.^58^ It has also been theoretically reported in the presence of pyrrolic N that the S dopant tends to dislocate from the graphene plane by approximately 1.32 Å to form the sp^3^ hybridization, whereas S-embedded pyridinic N-doped graphene retains the sp^2^ hybridization and showed the highest quantum capacitance even at a low S concentration.^55^

For the heterostructure of graphene/CNT, it has been shown that one can tune *C*_Q_ by choosing the structure to be defected. To be specific, the defected graphene–CNT hybrid exhibited a peak *C*_Q_ of 85.72 μF/cm^2^ at 0.4 V, whereas the pristine graphene-defective CNT hybrid exhibited a peak *C*_Q_ of 69.04 μF/cm^2^ at −0.4 V.^63^

### 4.5 van der Waals Heterostructure or Composites

At the beginning of this section, let us clarify the concepts and terminology used here. When an atom with a higher atomic size substitutes a carbon atom from a graphene matrix, for example, it is obvious that the foreign atom is protruding out from the in plane of graphene. There are other issues, such as the stability of the structure, strain in the structure, etc. Moreover, large-atom (like Cs with an atomic radius of 260 pm and Ti with an atomic radius of 140 pm) substitution in the graphene matrix (atomic radius of carbon is 70 pm), for example, may not be feasible experimentally. In the experimental point of view, most of the fabrication methods produce nanoparticle-decorated nanocarbon.^64^ For example, Zn-doped RGO is named in ref (65), where one can hardly see any experimental evidence of Zn doping (atomic radius of Zn is 135 pm) in the graphene matrix. Indeed, the existence of ZnO in the structure has been confirmed from the X-ray diffraction (XRD), energy-dispersive X-ray (EDX), and transmission electron microscopy (TEM) analyses.^65^ In this case, we prefer the terminology of “nanoparticle decoration”, “foreign atom adsorption”,^14^ or sometimes “heterostructure”.^66^

**Figure 11:**
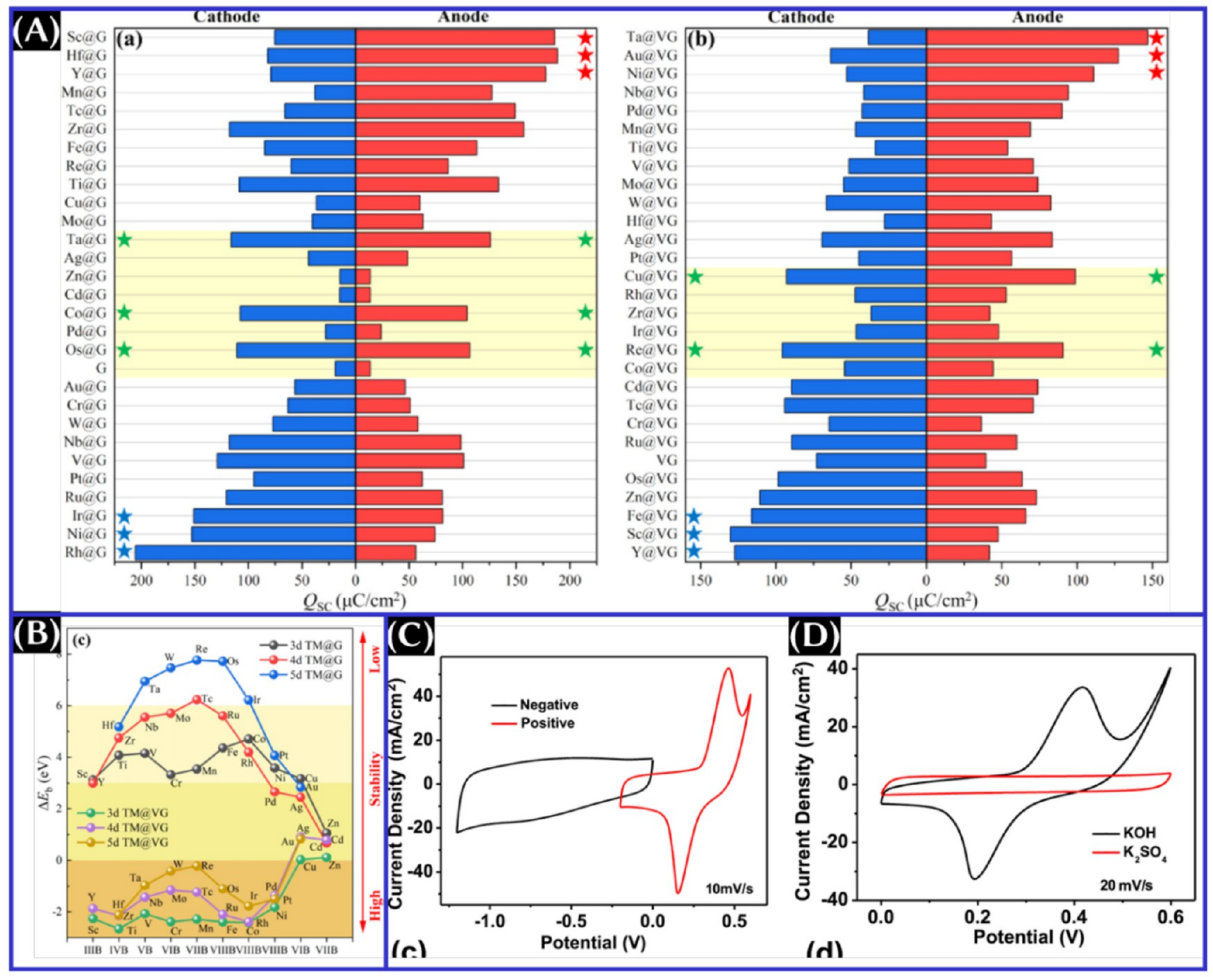
(A) Surface charge density and (B) structural stability of various metal-absorbed graphenes at positive and negative biases. These panels were reproduced with permission from ref (67). Copyright 2021 Elsevier, Ltd. Supercapacitive performance of the Ni nanoparticle-decorated CNT network (C) at a different potential window and (D) in different aqueous electrolytes. These panels were reproduced with permission from ref (71). Copyright 2017 Elsevier B.V.

Figure 11A summarizes the theoretical results of the quantum capacitance of nanoparticle-decorated graphene and vacancy-defected graphene.^54,67,68^ However, all the structures are not stable (Figure 11B).^67^ In some cases, like Zn@graphene or Zn@vacancy-defected graphene, *C*_Q_ is found to be lower than that of pristine graphene.^67^ However, when Zn is adsorbed on oxygenated graphene, it delivered 5.5 times higher *C*_Q_ than pristine graphene.^65^ It should be noteworthy to mention that the attachment of the Co_3_O_4_ nanocube is found to be more favorable than the decoration of the nanocube on graphene or the graphene surface with substitutional graphitic oxygen (G*).^69^ Moreover, the charge transfer for 3D GO/Co_3_O_4_ and 3D G*/Co_3_O_4_ is estimated to be 1.99e and 2.25e, respectively, which is higher than the other possible composites (1.15e for 2D G/Co_3_O_4_, 1.32e for 2D G*/Co_3_O_4_, 1.38e for 2D GO/Co_3_O_4_, 0.88e for 2D RGO/Co_3_O_4_, 1.73e for 3D G/Co_3_O_4_, and 1.13e for 3D RGO/Co_3_O_4_).^69^ Consequently, the maximum *C*_Q_ values obtained theoretically from 3D G*/Co_3_O_4_ and 3D G/Co_3_O_4_ are 862.81 and 880.34 F/g, respectively.^69^ This result again supports the necessity of a 3D film over the 2D film as an effective electrode material and suggests that forming a heterostructure on graphene with *in-plane* oxygen could be the better possible strategy to obtain a high charge storage property compared to the heterostructure on oxygen-functionalized graphene. However, we also emphasize that the structure, like Ni@G and Co@G, could be impressive to deliver higher theoretical *C*_Q_. There are many misleading experimental reports on these materials, where one can clearly see a battery-like feature instead of pseudocapacitance from them.^64,70^ Of course, one needs to choose a proper electrolyte and proper range of potential, where one can obtain pseudocapacitive behavior experimentally (panels C and D of Figure 11).^71^

### 4.6 2D Carbon Materials beyond Graphene

**Figure 12:**
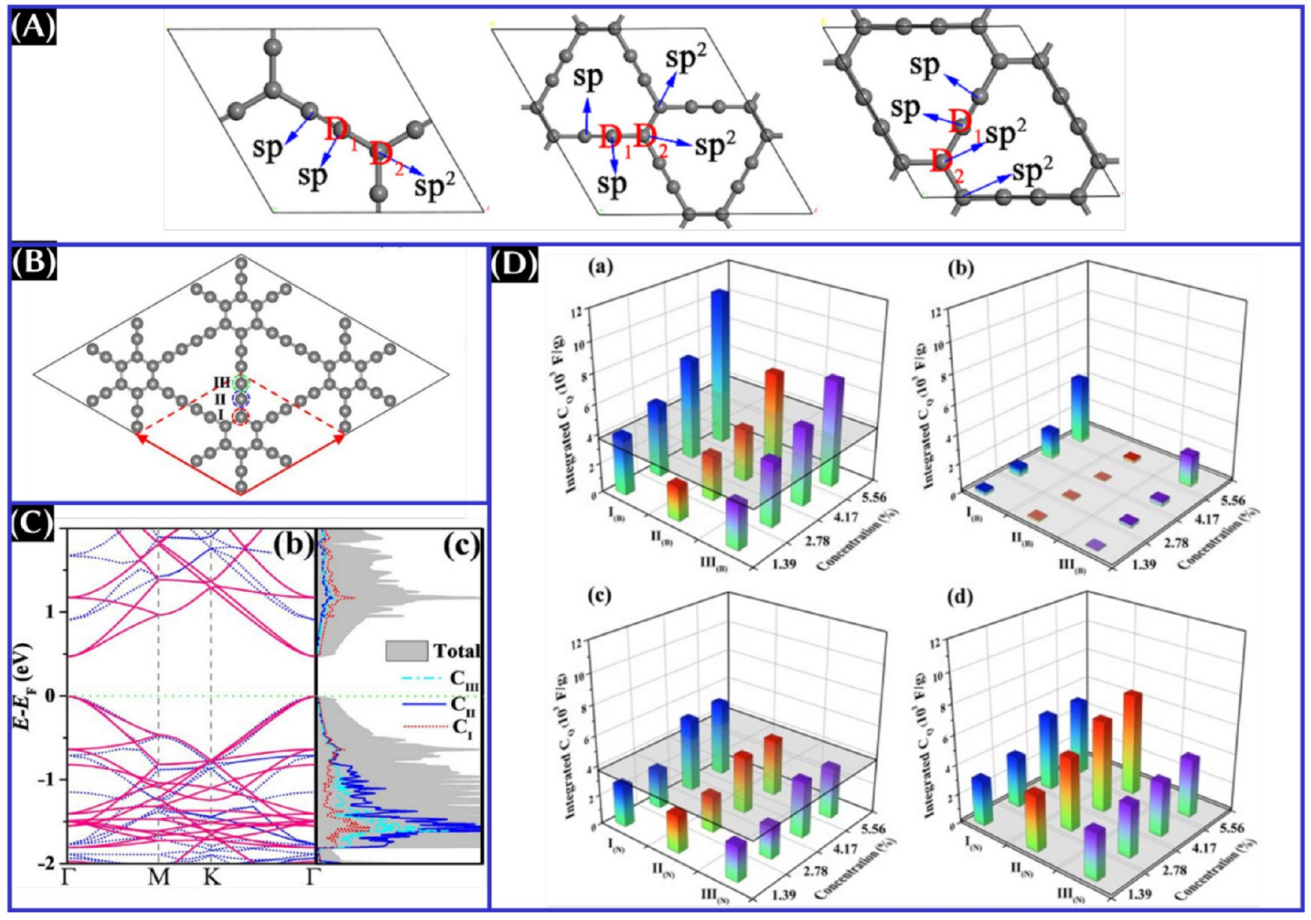
(A) Atomic arrangement of graphynes. This panel was reproduced with permission from ref (19). Copyright 2020 IOP Publishing, Ltd. (B) Atomic arrangement, (C) density of states, and (D) integrated *C*_Q_ of pristine and doped graphdiynes. These panels were reproduced with permission from ref (73). Copyright 2020 Elsevier B.V.

Besides graphene, there are emerging 2D carbon structures, such as graphynes and graphdiynes, that are formed by sp- and sp^2^-hybridized carbon atoms.^72^ In comparison to graphene, graphyne and graphdyne are anticipated as better charge storage electrodes theoretically. Eventually, the theoretical specific surface areas of α-, β-, and γ-graphynes are 5510, 4418, and 3457 m^2^/g, respectively, which are higher than that of graphene (2633 m^2^/g). It has been shown that not only is the specific capacitance of graphyne higher than that of graphene but also the quantum capacitance as well. The minimum *C*_Q_ values of α- and β- graphynes estimated using the DFT calculation are 78.7 and 541.3 F/g at 0 V, which are higher than that of graphene (42.6 F/g).^19^ The doping affects graphyne too, which depends upon the position of the dopants (Figure 12). Among the possible configurations, the maximum *C*_Q_ values obtained from B-doped α-graphyne, O-doped α-graphyne, and N-doped β-graphyne are 4531.6 F/g (0.26 V) at the D_1_ position, 4120.7 F/g (0.27 V) at the D_1_ position, and 1472.9 F/g (0.12 V) at the D_2_ position.^19^ It should be noted that the graphene structures are distorted when B and N are doped.

In the case of graphdiyne, simulated differential *C*_Q_ was 1805 F/g at −0.6 V, which is much higher than that of graphene (285 F/g). It has been simulated that differential *C*_Q_ of B-doped graphdiyne increases with the doping concentration and depends upon the position of the dopant. The B-doped graphdyine doped at site III with a doping concentration of 5.56% delivered the maximum theoretical differential *C*_Q_ of 4317 F/g.^73^ However, the most stable site of boron in the graphdiyne matrix is site I. Likewise, N-doped graphdiyne doped at site II with a concentration of 5.56% exhibited differential *C*_Q_ of 6150 F/g at 0.6 V. The theoretical report also estimated integrated *C*_Q_, because total storage capacity depends upon it, with the B-doped graphdiyne as the anode and N-doped graphdyine as the cathode being 10 557 and 6938 F/g at the doping concentration of 5.56%, respectively.^73^ For further enhancement, Cu adsorption on B/N-doped graphdyne presented a compelling advantage over the doping alone case.^14^ These high *C*_Q_ values are appealing and, thus, need further investigations.

As seen from Figure 12A, there is the presence of sp-hybridized carbon atoms with sp^2^-hybridized carbon atoms. It could be possible that the outstanding theoretical *C*_Q_ of graphyne may be due to the presence of sp carbon. Moreover, the appealing properties of sp-hybridized carbons include a very high theoretical surface area for H_2_ of 13 000 m^2^/g, Young’s modulus of 32 TPa, specific stiffness of 109 Nm/kg, thermal conductivity of 80 ± 26 kW m^–1^ K^–1^ at room temperature, and tunable electronic properties depending upon the bonding arrangement.^74−77^ Looking over it, the experimental investigation is carried out with a carbyne structure, and the areal capacitance of the supercapacitor device is found to be superior to many carbon nanostructures.^78^ There are also reports on sp-carbon-rich or carbyne-rich nanostructures as supercapacitor electrodes in three-electrode configurations.^79,80^ The maximum gravimetric (areal) capacitance obtained is 106.12 F/g (53.06 mF/cm^2^) for carbyne-enriched carbon anchored on nickel foam at a 5 mV/s scan rate in a 1 M Na_2_SO_4_ electrolyte,^79^ and the maximum areal capacitance is 0.32 mF/cm^2^ at 0.05 V/s for carbyne-rich nanostructured carbon in an ionic electrolyte.^80^ It is important to note that there is a major issue with the structural stability of as-synthesized carbyne.

## 5 Transition Metal (Di)chalcogenides

Layered 2D transition metal dichalcogenides have received significant attention for energy storage applications as a result of their high surface area, hydrophilic nature, variable oxidation states, high volumetric capacitance, etc.^2,81,82^ The general chemical formula of transition metal dichalcogenides is MX_2_, where M is the transition metals (Mo, W, Ti, Ta, Nb, V, etc.) and X is chalcogens (S, Se, and Te).

**Figure 13:**
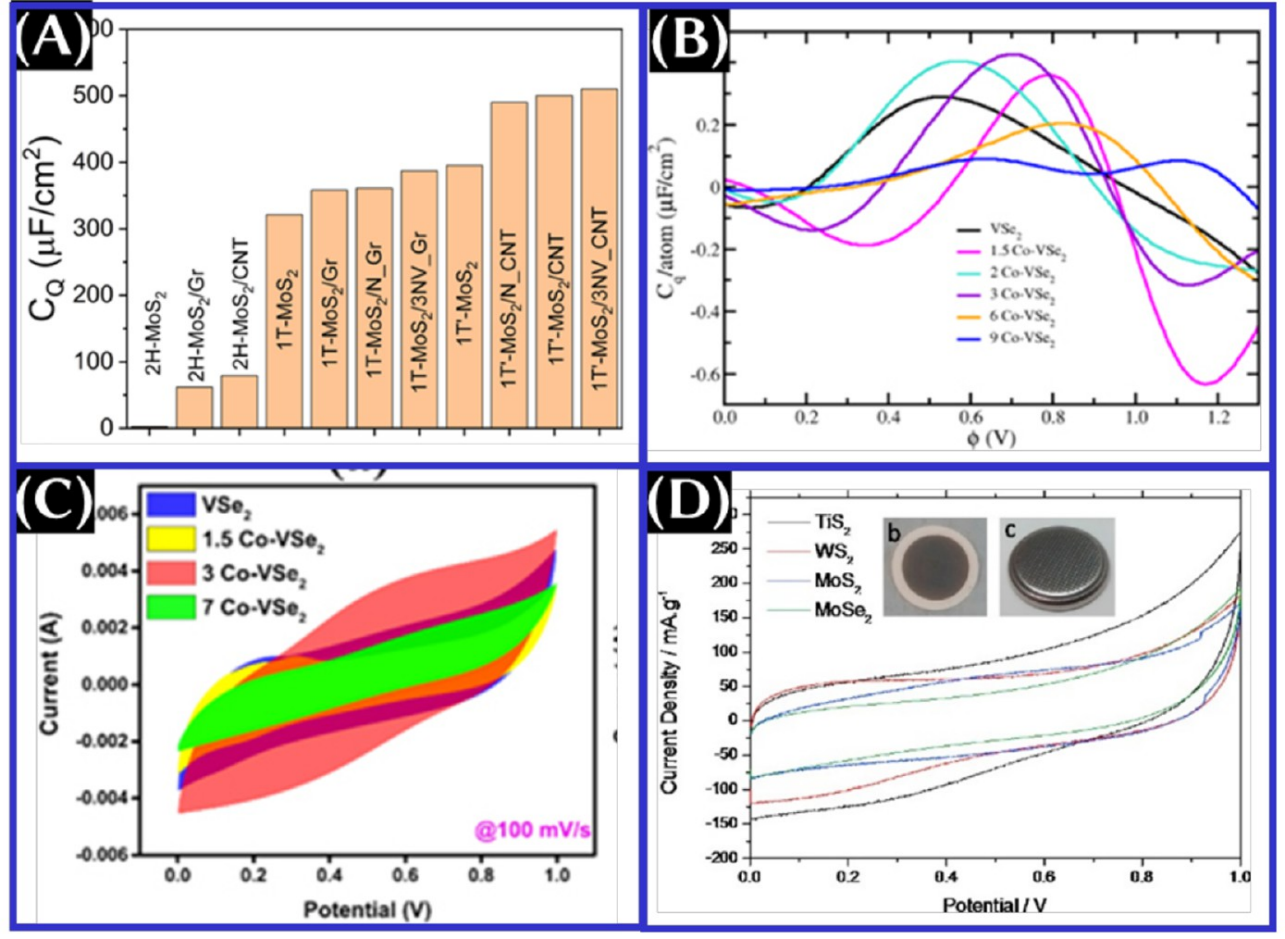
(A) Comparison of the theoretically estimated quantum capacitance value of MoS_2_-based structures. This panel was reproduced with permission from ref (18). Copyright 2021 Elsevier, Ltd. (B) Theoretically estimated quantum capacitance and (C) cyclic voltammogram of bare VSe_2_ and Co-doped VSe_2_. These panels were reproduced with permission from ref (85). Copyright 2022 IOP Publishing, Ltd. (D) Comparative cyclic voltammogram of TiS_2_, WS_2_, MoS_2_, and MoSe_2_ coin cell devices. This panel was adapted with permission from ref (86). Copyright 2016 Elsevier, Ltd.

Among the transition metal dichalcogenides, MoS_2_ is actively researched both theoretically and experimentally as a promising SC electrode as a result of its high volumetric capacitance of 400–700 F/cm^3^ for the metallic 1T phase compared to graphene (300 F/cm^3^). Other phases that exist are the semiconducting 2H phase, 3R phase, and distorted 1T′ phase. Among them, 1T-MoS_2_ is hydrophilic and much more electrically conducting (specifically 10^7^ times higher than the 2H phase). The hydrophilic surfaces are advantageous for a better electrode/electrolyte interaction, and good electrical conductivity is needed for excellent electronic transportation. The 2H phase shows a bandgap of 1.65 eV with zero DOS at the Fermi level, whereas both metallic 1T and 1T′ phases exhibit plenty of DOS around the Fermi level. Thus, *C*_Q_ is found to be higher for MoS_2_ with the metallic 1T phase (321 μF/cm^2^ at −0.6 V) and 1T′ phase (395 μF/cm^2^ at 0.6 V) than 2H-MoS_2_ (almost insignificant as a result of the absence of energy states near the Fermi level) (Figure 13).^18^ Distorted 1T′-MoS_2_ can be achieved by introducing carbon nanostructures (CNT or graphene) underneath, which induces localized strain effects leading to the distortion and phase transformation.^18^ In comparison to 1T′-MoS_2_/graphene (358 μF/cm^2^ at potential −0.6 V), 1T′-MoS_2_/CNT showed the enhanced DOS and, hence, theoretical *C*_Q_ of 500 μF/cm^2^.^18^ Moreover, heterostructures are not always profitable to have a higher *C*_Q_. For example, theoretical *C*_Q_ of MoS_2_/graphene is 0.28 μF/cm^2^ at 0 V, which was even lower than that of graphene (2.55 μF/cm^2^) and the MoS_2_ monolayer (65.22 μF/cm^2^).^83^ Of course, one can tailor the vacancy in MoS_2_/graphene, such as Mo, S, and C vacancies. S-vacancy MoS_2_/graphene showed a higher theoretical *C*_Q_ (273.98 μF/cm^2^) compared to its C vacancy (72.93 μF/cm^2^) and Mo vacancy (257.15 μF/cm^2^). The simulation result also shows that the maximum *C*_Q_ of Fe-doped Mo-vacancy defected MoS_2_/G was 346.99 μF/cm^2^ at a positive bias.^83^ On the contrary, it has also been reported that the Mo vacancy can change the electronic structure drastically compared to the S vacancy, such that a large amount of DOS was introduced, which, in turn, enhances *C*_Q_ to 209.733 μF/cm^2^.^84^

For the case of doping, the two common approaches include Mo substitution by transition metals or S atom replacement by group V (N, As, Sb, and Se) and group VII atoms (F and Cl). The maximum simulated *C*_Q_ is obtained for the N-substituted MoS_2_ monolayer (203.047 μF/cm^2^). However, there are no observable changes in the electronic states for the Se doping. For the group VII functionalization, *C*_Q_ is 139 and 252 μF/cm^2^ for F and Cl. In the case of Mo substitution, vanadium (V) could be the best choice because estimated *C*_Q_ is 263.721 μF/cm^2^ among Co, Cu, and Ni.^84^ It has been reported that substituting S by Al in single-vacancy MoS_2_ could be the choice for symmetric SC application and substituting S by B in pristine MoS_2_ could be the choice for asymmetric SC application among the Ti-, Au-, Ag-, Cu-, Al-, B-, N-, and P-doping pristine and single-vacancy MoS_2_ monolayer.^87^ Further enhancement of maximum *C*_Q_ and surface charge density can be achieved by increasing the doping concentration. It could be interesting to investigate the changes in electronic properties, DOS, and hence *C*_Q_ of the MoS_2_-based heterostructure with Mo substitution, Mo vacancy, and S substitution, simultaneously.

Another transition metal dichalcogenide, 1T-VS_2_ monolayer, showed high DOS near the Fermi level, and the highest simulated *C*_Q_ obtained is 377 μF/cm^2^ at +0.19 V. It can be enhanced further by introducing materials, like monolayer black phosphorus, underneath it via charge transfer from black phosphorus to VS_2_. As a result, the improved highest *C*_Q_ obtained is 428 μF/cm^2^ at +0.17 V.^88^ It is important to note that monolayer black phosphorus does not have DOS near the Fermi level. Doping is another adoptable strategy for V-based dichalcogenides. However, beyond the 3% level, Co doping of 1T-VSe_2_ shows adverse effects on the quantum capacitance and gravimetric capacitance (panels B and C of Figure 13). While the experimental results are compared for total gravimetric capacitance of different transition metal dichalcogenides (Figure 13D), the trends of capacitance value follow as TiS_2_ (4.60 F/g) > WS_2_ (3.50 F/g) > MoS_2_ (3.40 F/g) > MoSe_2_ (2.57 F/g) at a 10 mV/s scan rate in a 1 M Na_2_SO_4_ aqueous electrolyte. The MoS_2_ cell exhibits the lowest phase constant of 60° at a low frequency compared to that of TiS_2_ (75°). The higher capacitance value of TiS_2_ is attributed to the low density and higher electrical conductivity.^86^

FeS is another emerging material that can be considered as an energy storage electrode. *C*_Q_ of hexagonal FeS is estimated from first-principles calculations to be 408 F/g. When the vacancy is introduced in the structure, *C*_Q_ enhanced to 2280 F/g at a positive bias.^89^

## 6 MXene

MXenes have recently emerged as promising 2D materials of transition metal carbide/nitride/carbonitride in the field of energy storage and other suitable applications as a result of the features of metallic conductivity, large interlayer spacing, easy functionalization, and redox-active surface oxide-induced pseudocapacitive behavior.^2,90^ The general formula for MXenes is M_*n*+1_X_*n*_T_*x*_, where M, X, and T_*x*_ stand for the transition metal, carbon and/or nitrogen, and surface terminations with *n* = 1–4, respectively. Some of the most researched and significantly attracted MXenes are Ti_3_C_2_T_*x*_, Ti_2_CT_*x*_, V, and Nb. Importantly, the volumetric capacitance of Ti_3_C_2_T_*x*_ MXene hydrogel obtained is 1500 F/cm_3_ with respect to Hg/HgSO_4_ at 2 mV/s in H_2_SO_4_, which is much higher than that of MoS_2_ or graphene.^22^

**Figure 14:**
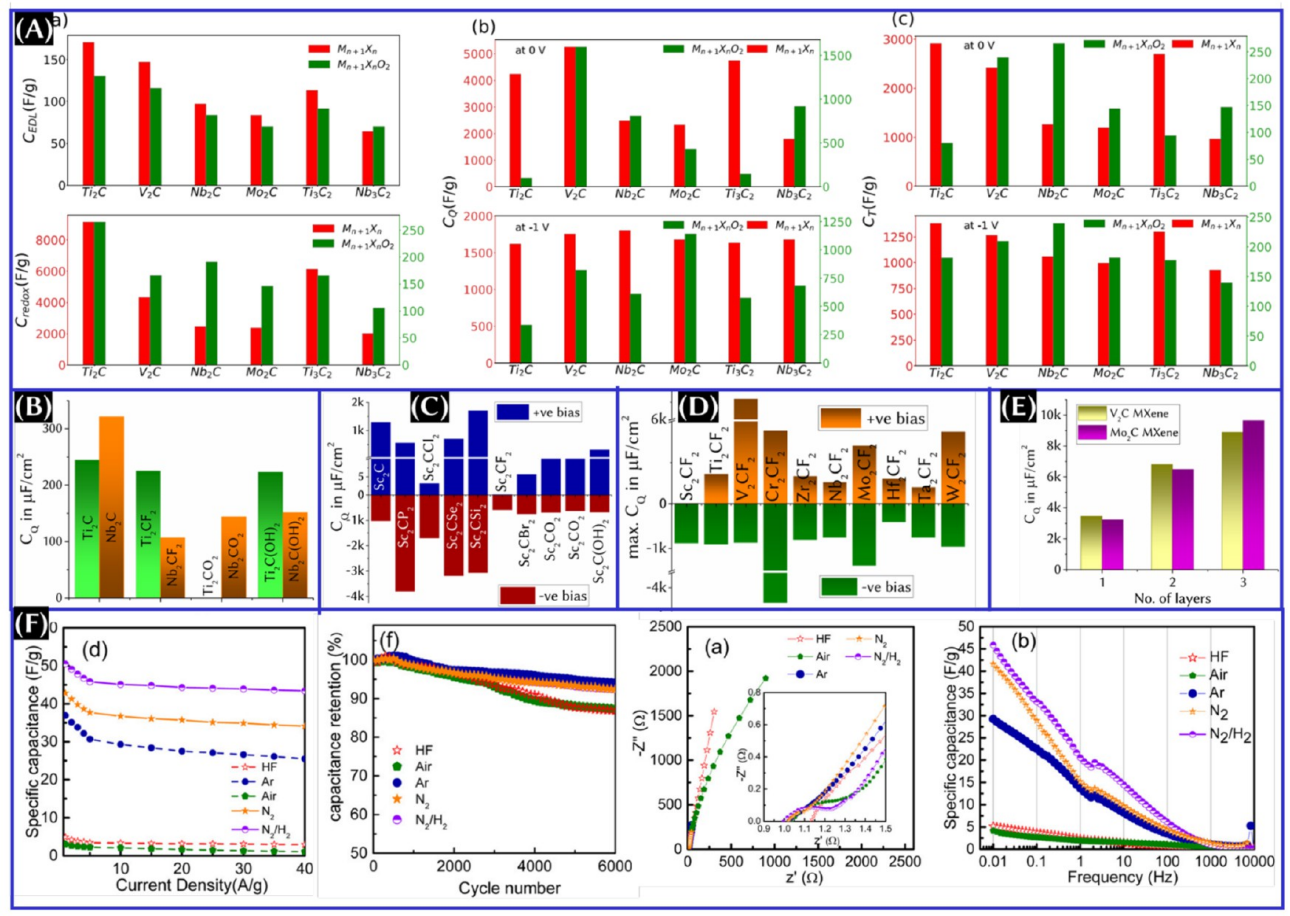
(A) Double layer, quantum capacitance, and total capacitance of bare and oxygen-functionalized MXenes. This panel was reproduced with permission from ref (91). Copyright 2021 IOP Publishing, Ltd. Theoretical quantum capacitance of (B) functionalized Ti_2_C and Nb_2_C MXenes,^94^ (C) functionalized SC_2_C MXene with different termination groups,^95^ (D) MXene with a F-terminated group,^96^ and (E) V_2_C and Mo_2_C MXenes with respect to the number of layers. Data for panels B–E are either taken from the text/table or extracted from the corresponding references using the WebPlotDigitizer software authored by Ankit Rohatgi. (E) Experimental charge storage performance of the modified Ti_3_C_2_T_*x*_ MXene/KOH electrolyte symmetric cell. This panel was reproduced with permission from ref (97). Copyright 2015 American Chemical Society.

In contrast to the pristine carbon-based materials, MXenes generally show higher values of the DOS and *C*_Q_. Figure 14A^91^ summarizes the contribution from EDLC, redox capacitance, *C*_Q_, and total capacitance (*C*_T_) of Ti-, V-, Nb-, and Mo-based MXenes. To calculate theoretically, a few assumptions made in ref (91) are MXene being negatively charged, H^+^ ion adsorption on the electrode surface, and *C*_Q_ and *C*_T_ measuring at 0 and −1 V because they have two extrema close to those two voltages. The total capacitance calculated theoretically is mostly in good agreement with the experimentally obtained result. MXenes commonly feature the functional/terminated groups (denoted as T_*x*_) on the surface. On the basis of the synthesis techniques, T_*x*_ can be different. For example, T_*x*_ is O, OH, Cl, and F for chemical etching,^92^ O and OH for hydrothermal, and Cl or halides with O-functional groups for molten salt techniques. Oxygen functional groups are considered unavoidable because of the chemical process involved in all techniques, and they are responsible for aiding the pseudocapacitance. Eventually, the theoretical calculations on *C*_Q_ and surface charge density suggested V_2_CT_2_ MXene with mixed terminations as a suitable anode material of asymmetric supercapacitors in aqueous and ionic/organic systems.^93^ The result obtained theoretically for pristine and functionalized MXenes (Figure 14) can be summarized as follows:^91^ (i) Ti_2_C has the highest EDLC and *C*_T_, even compared to Ti_3_C_2_, whereas V_2_C has a higher *C*_Q_ at 0 and 1 V, and Nb_2_C has the highest *C*_Q_ at 1 V among the studied pristine MXenes. (ii) O functionalization lowered the EDLC, *C*_Q_, and *C*_T_ for all MXenes.^94^ (iii) Among the functionalized MXenes, O-functionalized V_2_C has a higher *C*_Q_ at 0 V and O-functionalized Mo_2_C has the highest *C*_Q_ at 1 V. However, O-functionalized Nb_2_C has the highest total capacitance at both 0 and 1 V.

Instead of the O-functional group, the charge storage capacity can be enhanced with the replacement of carbon in a titanium octahedron by oxygen in Ti_3_C_2_T_*x*_ MXene. It has been reported that O-doped MXene synthesis from the O-functionalized MAX phase is a more facile approach, called *in situ* oxygen doping.^98^ From the O doping, the DOS near the Fermi level is improved, which is reflected in the enhanced interfacial capacitance of O-doped Ti_3_C_2_T_*x*_ compared to its bare counterpart.^98^ In this way, one can decrease the valence state of the inner Ti atoms, which promotes electronic transport, and increase the valence state of outer Ti atoms, which is responsible for the enhanced pseudocapacitance.

The above result (Figure 14A) is based on only the O-functional or termination group. On the basis of the synthesis procedure, as mentioned above, there are other functional groups. Studies reveal that, like O-functional groups, the existence of other functional groups also has similar adverse effects on the Ti_2_C and Nb_2_C MXenes.^96^ As seen from Figure 14B, along with −OH, −F also has the least impact on *C*_Q_ for Ti_2_C MXene.^99^ Despite the lower *C*_Q_ values of V_2_C and Mo_2_C caused by the introduction of functional groups, the functionalized MXenes still have a higher *C*_Q_ compared to the other electrode materials.^96^ There is also an exception for Sc_2_C MXene with other functional groups as well. The maximum differential *C*_Q_ of Sc_2_CSi_2_ with T of P, Cl, Se, and Si is higher than that of monolayer Sc_2_C in both aqueous and organic electrolytes (Figure 14C).^95^

In reality, after the synthesis, MXenes possess multiple termination groups, and these are unavoidable. Theoretical results suggest that removing the functional groups from the surface of electrode materials could be a better option to obtain a high energy storage performance. From the experimental side, on the basis of testing in a two- or three-electrode configuration, it is also observed that post-annealing as-synthesized MXene has better performances. The alkalinized (by KOH treatment) and annealed (under Ar at 673 K) Ti_3_C_2_T_*x*_ film exhibits higher gravimetric (volumetric) capacitance of 543 F/g (2063 F/cm^3^) at 1 A/g with 98% capacitance retention after 8000 cycles, which is higher than the untreated Ti_3_C_2_T_*x*_ film (281 F/g).^100^ Among the post-annealing under different environments (air, Ar, N_2_, and N_2_–H_2_), Ti_2_CT_*x*_ MXene annealed at N_2_–H_2_ provides the best storage properties compared to as-synthesized MXene.^97^ In both cases,^97^^100^ the improvement in performances is mainly attributed to the increased surface area, increased interplanar distance, increased crystalline order, highest carbon content, and removal of −F and −OH terminal groups. On the contrary to F removal, the *C*_Q_ value obtained theoretically from F-functionalized MXene is quite impressive.^93^ Ca-adsorbed Ti_3_C_2_F_2_ and Li-adsorbed Ti_3_C_2_F_2_ showed the highest *C*_Q_ of 488.153 and 259.490 μF/cm^2^, respectively, among the all of possible combinations.^99^ We emphasize that the use of F is not recommended because it reduces the electrochemical reactivity and electrical conductivity of MXene, while it is also hazardous and causes some safety issues.

The harsh synthesis techniques of MXene from its parent structure leave an atomic vacancy in the MXene structure, which has a significant impact on the diffusion behavior of electrolyte ions (Li^+^, Na^+^, K^+^, etc.). The (opto)electronic properties of the final structure obviously depend upon the vacancy type.^101^ It has been predicted that *C*_Q_ of Ti_2_CO_2_ can be enhanced at a positive potential further by increasing the oxygen vacancy concentration because a larger charge transfer takes place for neighboring O and Ti atoms. It is also important to note from the theoretical study that one oxygen vacancy is more effective compared to two or three oxygen vacancies.^102^ Ti_2_CO_2_ with oxygen vacancy concentrations of 11.11 and 16.67% has a lower maximum *C*_Q_ of 3131.18 and 3573.14 μF/cm^2^ at a positive potential, respectively. Apart from the O vacancy, the C or M vacancy also enhances the DOS at the Fermi level. As predicted, Zr_2_CO_2_ MXenes with Zr vacancy can be chosen as cathode materials, whereas Zr_2_CO_2_ MXenes with C and O vacancies can be a good choice for anode materials.^101^

Apart from the termination groups, doping is another approach for MXene as well to change the DOS and, hence, *C*_Q_.^15,103^ The dopants apparently determine the intrinsic properties of the final structure.^15^ To increase *C*_Q_ further, two cobalt (Co) atoms were doped, and the increased *C*_Q_ was attributed to the increased DOS contribution from 3d and 4s electrons of Co. N doping into carbide-based MXene could be another alternative approach to enhance *C*_Q_.

We stress that, apart from the carbide-based MXene, there are also nitride-based MXenes. It could be interesting to evaluate the superiority between N-doped carbide and nitride MXenes. Eventually, in contrast to Nb_2_C MXene, niobium nitride MXene emerges as promising electrode material. We highlight that simulated *C*_Q_ of NbN at negative and positive biases is found to be 834.5 and 1683.7 F/g compared to Nb_4_N and Nb_5_N_6_.^104^ The maximum *C*_Q_ has been observed for Nb_2_N (1196.28 μF/cm^2^ at −1 V and 844.8 μF/cm^2^ at 0.5 V) and Nb_4_N_3_ (174.86 μF/cm^2^).^105^ Remarkably, increasing the number of layers has profound effects on enhanced *C*_Q_ for both Nb_2_N and Nb_4_N_3_.

## 7 Other 2D Materials

There are many 2D materials, apart from graphene, transition metal chalcogenides, and MXenes, in the pipeline, and they have remarkable features. They are sometimes even found to be superior to graphene based on the theoretical calculation. *C*_Q_ of silicene is predicted to be 2 times higher than that of graphene, even at a low bias.^106^ To improve *C*_Q_ of these materials, the strategy is similar to graphene, namely, doping, introducing defects, etc. Among these, silicene with a six vacancy-defected structure performed better at the voltage of 0–0.5 V.^107,108^ It is reported on the basis of the theoretical calculation that the co-doping with the transition metal and nitrogen to silicene delivered higher charge storage and *C*_Q_ compared to the single doping with the transition metal or N. The DOS contribution near the Fermi level only comes from the 3d state of the transition metal atom and the 3p state of the Si atom, whereas N helps to stabilize the system. The higher the N concentration, the more stable the structure.^109^ Theoretical *C*_Q_ of germanene (3.51 μF/cm^2^) is also found to be higher than that of graphene at 0 V. Although doping can have a significant effect on DOS, this is not always the case. For example, N-doped germanene showed a theoretical *C*_Q_ of 45.32 μF/cm^2^ at 0.01 V, and Ti-doped germanene exhibited the highest *C*_Q_ of 91.47 μF/cm^2^ at 0.2 V compared to Cr, Co, and Mn doping,^108^ whereas boron and aluminum doping does not show any significant enhancement. There are other 2D materials, like arsenene^110^ and δ-6 borophene,^111^ and theoretical studies have been conducted focusing on tailoring the electronic properties, DOS, and hence *C*_Q_ by means of doping, defect, or heterostructure formations.

As seen from the above literature analysis, the major issues of these 2D materials as an energy storage electrode include (i) mass-scale production of these 2D materials and, in particular, synthesis of the freestanding film, which remains a challenge, (ii) material stability, and hence (iii) very limited studies on the applications because there is a lack of experimental confirmation of theoretical predictions.^112^

## 8 Temperature Dependency

**Figure 15:**
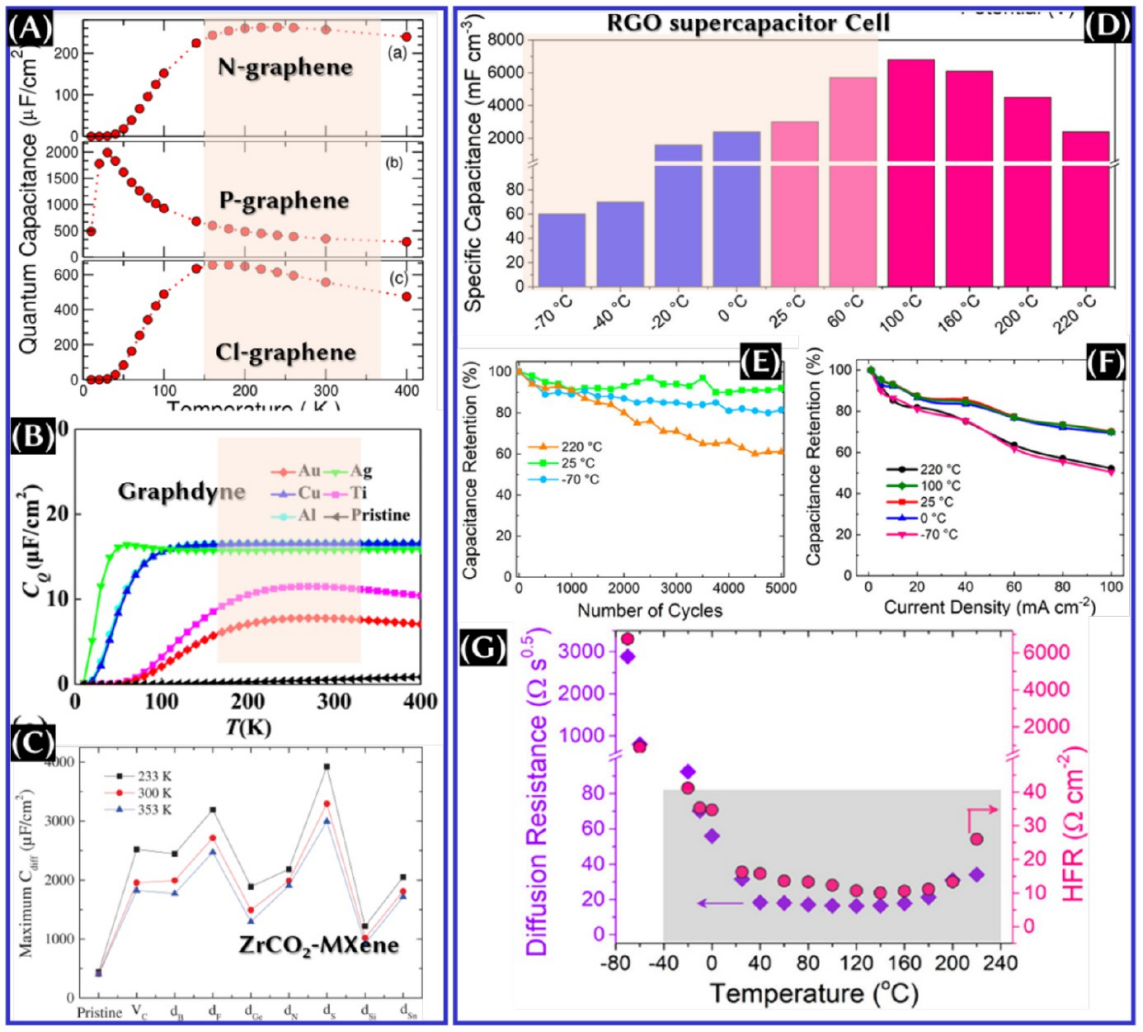
Theoretical quantum capacitance variation of (A) graphene,^13^ (B) graphdyne,^14^ and (C) ZrCO_2_ MXene^15^ with respect to the temperature. These panels were reproduced with permission from refs (13−15). Copyrights 2019 IOP Publishing, Ltd., 2023 Royal Society of Chemistry, and 2021 Wiley Periodicals LLC, respectively. (D) Volumetric capacitance, (E) capacitance retention, (F) rate performance, and (G) changes in diffusion resistance and high-frequency resistance of the RGO symmetric cell. This panel was reproduced with permission from ref (113). Copyright 2020 American Chemical Society.

This section discusses the temperature dependence of the quantum capacitance. Most of the simulations and even experimental testing as supercapacitor electrodes are carried out at room temperature. However, supercapacitors should be capable of working in the range from −100 °C (173 K) to 60 °C (343 K), with extremely low failure rates. Because the quantum capacitance is an intrinsic property of electrode materials, it is interesting indeed to see what happens to *C*_Q_ of electrodes at various temperatures. Considering pristine graphene-based materials, structures are theoretically found to be quite stable at elevated temperatures theoretically. However, once functionalized or doped, structural properties change (Figure 7D). Eventually, drastic changes can also be seen in *C*_Q_ with respect to the temperature (Figure 15), which is attributed to the Kondo effect.^13^ This effect is confirmed by a spin-polarized DFT study that doped adatoms show magnetic behavior and there is localization of DOS near Fermi energy.^13^ In the case of graphdiyne, no significant changes in *C*_Q_ have been observed,^14^ whereas metal-adsorbed graphdiyne shows increasing trends of *C*_Q_ up to a certain temperature and then becomes saturated. We assign the temperature where it saturates as the cutoff temperature. The cutoff temperature is found to be different for the different metals adsorbed on the surface of graphdiyne (Figure 15B).^14^ In the case of ZrCO_2_, *C*_Q_ of pristine MXene has almost no impact on the temperature. However, *C*_Q_ becomes lower and higher at lower and higher temperatures with respect to room-temperature *C*_Q_, respectively, and this effect does not depend upon whether it is vacancy-defected or heteroatom-doped.^15^ Within the temperature region of interest (from −100 to 60 °C or from 173 to 343 K), as highlighted by the shadowed color in panels A, B, and D of Figure 15, *C*_Q_ of the electrode has an almost decreasing trend with the temperature. We also emphasize the changes in the electrode and/or device that happen in the performance from real test results (panels D–G of Figure 15).^113^ In terms of the total capacitance of the cell, it is obvious to observe that the higher electrolyte ion mobility (even if there is the possibility of evaporation and freezing of ionic movement at respective higher and lower temperatures), the improved electrical conductivity of the electrode at higher temperatures, which is, in turn, higher specific capacitance, better coulombic efficiency, and higher specific energy/power density of the electrode and the cell up to a certain temperature. There is also the possibility of the phase change of the SC electrodes made of metal-oxide-type materials with the temperature operation, and sometimes, it cannot be reversible.

## 9 Electrolyte Dependency

**Figure 16:**
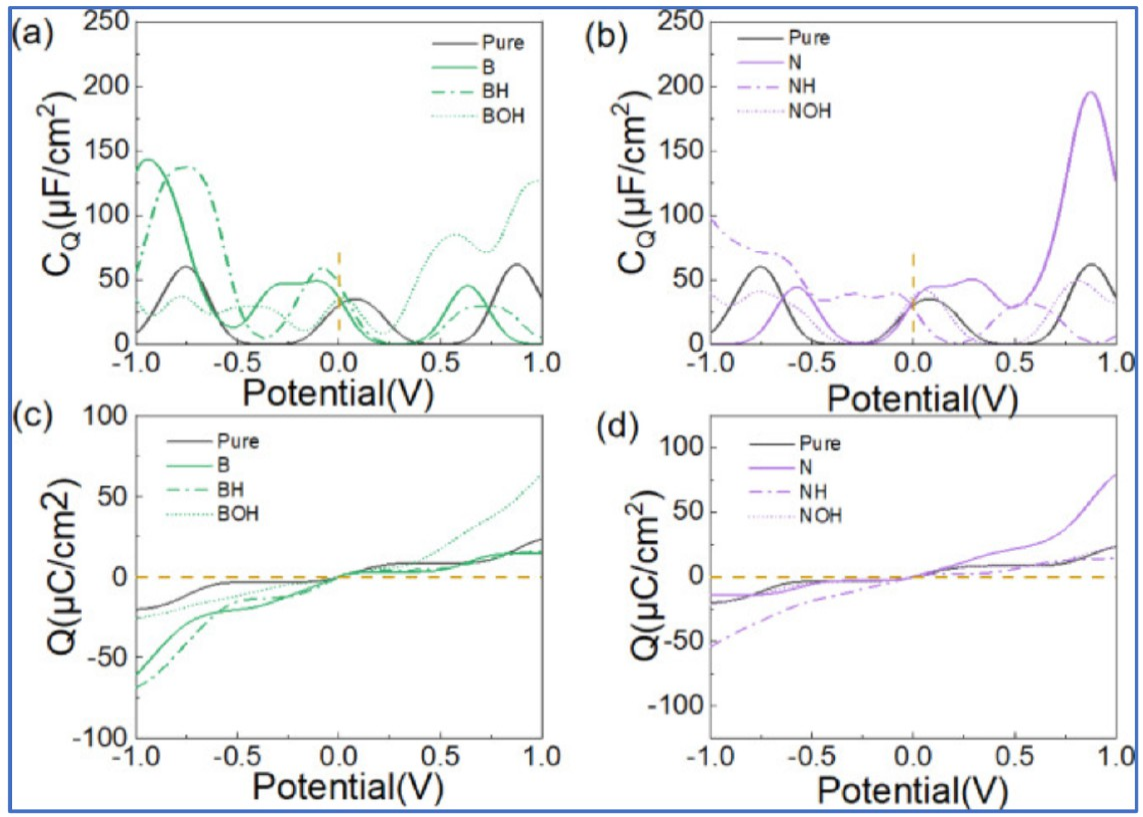
Theoretical quantum capacitance of CNT and doped CNT in acidic (H) and alkaline (OH) electrolyte media. These panels were reproduced with permission from ref (16). Copyright 2022 Elsevier B.V.

This section explores the dependence of *C*_Q_ upon specific electrolytes. The expected answer is certainly not because *C*_Q_ is an inherent property of the electrode. First-principles calculations on the desolvation behavior of Li^+^, Na^+^, and K^+^ at the edge plane pores and basal plane pores of porous carbon materials confirmed that there are no significant changes in the total DOS near the Fermi level.^114^ On the other hand, a recent theoretical study revealed that the maximum *C*_Q_ was found for the B-doped CNT in the alkaline electrolyte at a positive bias, the B-doped CNT in the acidic electrolyte at a negative bias, and the N-doped CNT in the alkaline electrolyte at a negative bias (Figure 16).^16^ Likewise, *C*_Q_ of 1T-TaS_2_ is found to be enhanced with K^+^ and Na^+^ ion intercalation and lowered with Li^+^ ion intercalation. Moreover, the highest theoretical *C*_Q_ is found for 2H-TaS_2_ intercalated with Li^+^ ions and 3R-TaS_2_ with K^+^ ion intercalation.^17^ Eventually, the alkali ion intercalation into 2H- and 3R-phase MoS_2_ transformed them into the stable metallic 1T-MoS_2_ phase.^115^ Moreover, despite higher *C*_Q_ values obtained from K^+^ and Na^+^ ions, the reversibility is maintained in the case of Li-ion intercalation. For the overall best performance from the electrode, the Li^+^/Na^+^ co-intercalation is proposed. These results reveal that the *C*_Q_ values of the electrode depend upon the electrolyte used, and it is recommended to choose the appropriate electrolyte for the specific electrode.

## 10 How To Measure *C*_Q_ Experimentally

**Figure 17:**
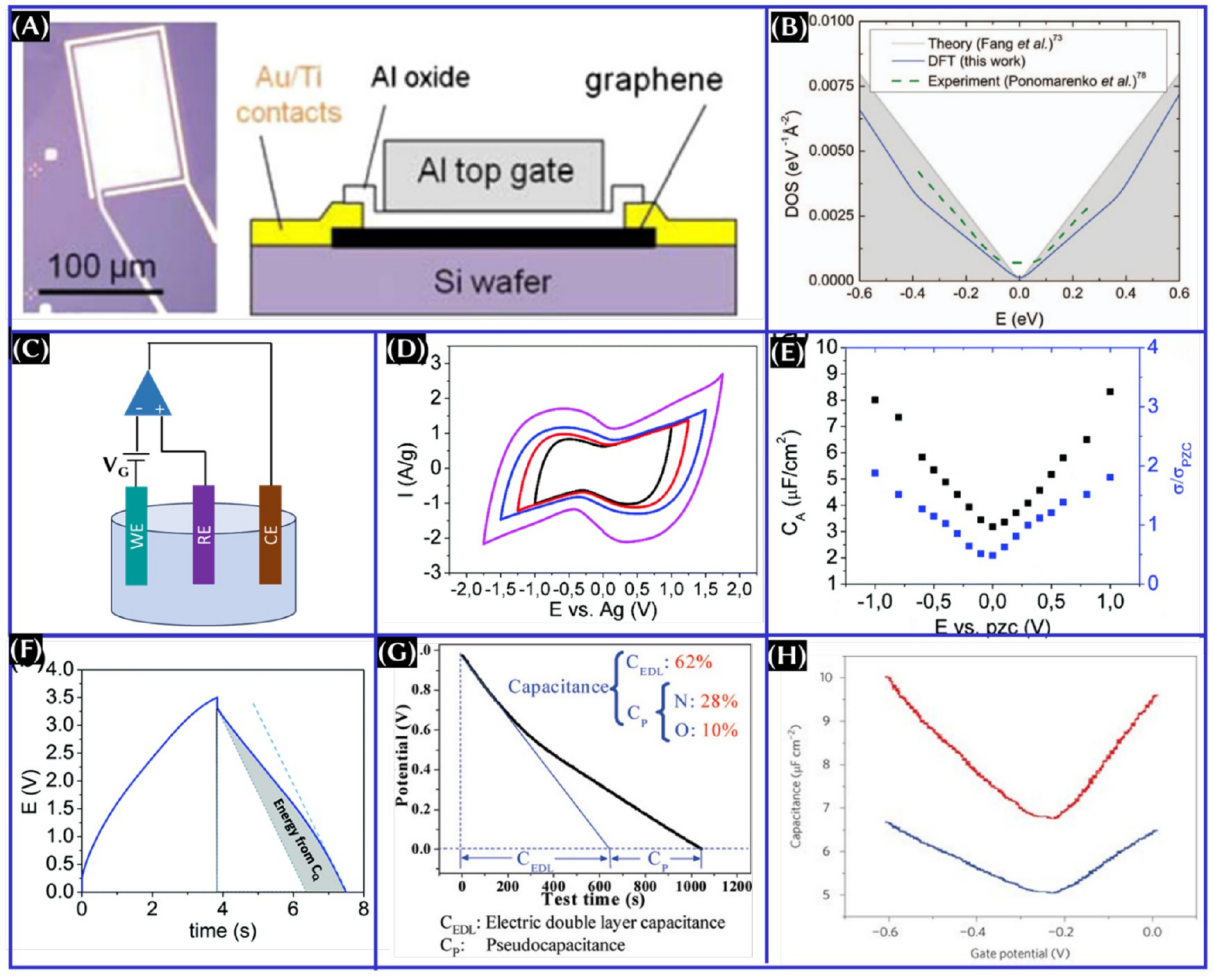
(A) Schematic of the graphene–Al_2_O_3_–Al capacitor. This panel was reproduced with permission from ref (117). Copyright 2010 American Physical Society. (B) Comparison of density of states of graphene from theory, experiment, and DFT. This panel was reproduced with permission from ref (28). Copyright 2012 IOP Publishing, Ltd. (C) Schematic of the quantum capacitance measurement setup for the graphene electrode.^118^ (D) CV at 50 mV/s at different voltage limits, (E) area-normalized capacitance and normalized conductivity versus potential at zero charge obtained from electrochemical impedance at 10 mHz and different bias voltages, and (F) charge–discharge profile of the symmetric device made with macroscopic fibers of CNTs as electrodes and 1-butyl-3-methylpyrrolidinium bis(trifluoromethylsulfonyl)imide (PYR14TFSI) as the ionic liquid electrolyte used. These panels were reproduced with permission from ref (119). Copyright 2016 Royal Society of Chemistry. (G) Capacitive contribution from nitrogen-enriched nanocarbons with a 3D continuous mesopore structure. This panel was reproduced with permission from ref (120). Copyright 2010 American Chemical Society. (H) Total capacitance (blue line) and quantum capacitance (red line) of graphene can be measured with the measurement setup presented in panel C. This panel was reproduced with permission from ref (118). Copyright 2009 Nature Publishing Group.

The concept of quantum capacitance was first introduced by Luryi for 2D electron gas in a quantum well, by neglecting the screening effect that occurred for metals.^116^ From the experimental point of view, of course, one can obtain the quantum capacitance by measuring the applied gate voltage from Hall measurements (Figure 17A). The discrepancy between the theoretical and experimental results (Figure 17B) near 0 V is attributed to the oxide layer thickness, defects present in graphene, and graphene–oxide surface interaction.^28^ Here, we highlight that the meaning of oxide is the SiO_2_ layer/Si (most used substrate for the Hall effect measurements). In this review, our concern is the estimation of *C*_Q_ of the electrode when the electrochemical energy storage device testing is conducted under a defined electrolyte (Figure 17C).

The voltage-dependent butterfly-shaped cyclic voltammetry (CV), a common behavior of the quasi-metallic nature of carbons, corresponds to the quantum capacitance (Figure 17D). The quasi-metallic behavior of the electrode can also be ascertained from the plot of the area-normalized capacitance and longitudinal conductivity versus the applied potential (Figure 17E). The data presented in Figure 17D are obtained from the impedance measurements in the three-electrode cell with respect to the potential at zero charge (pzc). The quantum capacitance contribution can be calculated from the charge–discharge profile by plotting a discharge line with the slope at pzc (Figure 17F).^119^ However, the extended line of the discharge profile is also used to estimate the pseudocapacitive contribution for the symmetric cell made with nitrogen-enriched nanocarbons with a 3D continuous mesopore structure (Figure 17G).^120^ It could be interesting to estimate the EDLC, *C*_Q_, and pseudocapacitive contributions from the discharge profile of doped carbon or any other structure by compiling the estimation procedure mentioned in refs (119 and 120). In another method, a gate voltage can be applied to the electrode/electrolyte system (Figure 17C) to obtain the total capacitance from a V-shaped capacitance–gate voltage curve in the electrolyte with respect to the reference electrode (blue line in Figure 17H). To measure the value of *C*_Q_ (red line in Figure 17H), one can subtract the total capacitance by *C*_dl_. *C*_dl_ is basically measured from the three-electrode test using the cyclic voltammogram within the scanning range of the linear region, where only non-faradic activity occurs,^121^ or using the impedance spectroscopy.^7^ Alternatively, *C*_dl_/*A* can be calculated using eq 1.^118^

## 11 Summary, Challenges, and Outlook

### 11.1 Summary

Supercapacitors or electrochemical capacitors are promising energy storage devices that provide more charge storage capacity than conventional capacitors and higher cycle life and higher power density than the battery. The electrode material of the energy storage device plays a key role, and the total charge storage capacitance contribution comes from the electrode/electrolyte interactions and quantum capacitance of the electrode (*C*_Q_). *C*_Q_ is connected in series with *C*_EE_ (capacitance as a result of the electrode/electrolyte interaction). In summary, we have discussed the quantum capacitance of various electrode materials, namely, carbon, 2D materials, and their composites, and hence, it has a significant impact on the total capacitance. Table 3 summarizes the theoretical calculations of *C*_Q_ of carbon and 2D materials. Besides the knowledge of the surface engineering of the electrode, electrolyte modification, and electrode/electrolyte interaction improvements, the enhancement of *C*_Q_ became equally important to obtain high storage performance from the fabricated supercapacitor device. By tuning the density of states by introducing defects, dopants, or other heterostructures, one can enhance the quantum capacitance of the electrode materials and the potential window of the energy storage device. Dependent upon the performance of the electrode and its quantum capacitance behavior, one can choose the right material and the best approach for its potential applications, because all strategies may not be leading to the enhanced *C*_Q_. The electrode material with a high *C*_Q_ value in the positive bias and a low *C*_Q_ value in the negative bias window is a suitable choice as an anode of an asymmetric supercapacitor. Meanwhile, the electrode materials with high and low *C*_Q_ values in the negative bias and negative bias window, respectively, can be a choice as cathode materials for asymmetric supercapacitors.^68^ One can also measure the surface charge density at positive bias (*Q*_a_) and negative bias (*Q*_c_). The value of *Q*_a_/*Q*_c_ can be an indication of the choice of the electrode.^67^ In general
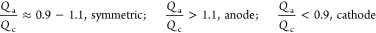
Moreover, *C*_Q_ of the electrode also has a dependency upon the temperature of operation as the surface charge density, and DOS depends upon the temperature. It has also been seen that *C*_Q_ of the electrode can be tailored by the intercalation of different electrolyte ions; this result can guide experimentalists to choose the right electrolyte for the desired electrode materials.

**Table 3:** Quantum Capacitance of Carbon and 2D-Material-Based Supercapacitor Electrodes Based on the Theoretical Calculations^a^

electrode material		dopant(s)/metal adsorbed/ion intercalated	quantum capacitance, *C*_Q_	method of determination/approach
graphene^38^		pristine	2.55 μF/cm^2^ at 0 V	*ab initio* density functional theory (DFT)
		vacancy defect	44.38 μF/cm^2^ at 0 V	
		Stone–Wales defect	120.72 μF/cm^2^ at 0 V	
		silicon (Si)	169.76 μF/cm^2^ at −0.29 V	
			49.02 μF/cm^2^ at 0.11 V	
		aluminum (Al)	113.73 μF/cm^2^ at −0.39 V	
			79.89 μF/cm^2^ at 0.06 V	
		phosphorus (P)	76.73 μF/cm^2^ at −0.3 V	
			56.07 μF/cm^2^ at 0.45 V	
		boron (B)	112.52 μF/cm^2^ at 0 V	
		Al + Stone–Wales defect	102.61 μF/cm^2^ at −0.38 V	
		B + Stone–Wales defect	76.18 μF/cm^2^ at −0.42 V	
		P + Stone–Wales defect	59.36 μF/cm^2^ at 0.12 V	
		sulfur (S) + Stone–Wales defect	88.31 μF/cm^2^ at −0.46 V	
graphyne^19^		α-graphyne	78.7 F/g at 0 V (42.6 F/g for graphene)	*ab initio* DFT
		β-graphyne	541.3 F/g at 0 V	
		B-doped α-graphyne	4531.9 F/g at 0.26 V	
		B-doped β-graphyne	3626.2 F/g at 0.25 V	
		B-doped γ-graphyne	3587.7 F/g at 0.16 V	
		N-doped α-graphyne	1196.3 F/g at 0.48 V	
		N-doped β-graphyne	1472.9 F/g at 0.12 V	
		N-doped γ-graphyne	1221.1 F/g at −0.06 V	
		O-doped α-graphyne	4120.7 F/g at 0.27 V	
		O-doped β-graphyne	1417.7 F/g at −0.02 V	
		O-doped γ-graphyne	1586.5 F/g at −0.60 V	
graphdyne^73^		pristine	1805 F/g at −0.6 V (264 F/g for graphene)	DFT-based first-principles calculations
		5.56% B	4317 F/g at −0.3 V	
		5.56% nitrogen (N)	6150 F/g at 0.6 V	
single-walled carbon nanotube^122^		Sc	52.58 μF/cm^2^ at −0.6 eV	*ab initio* spin-polarized DFT
		Cr	43.21 μF/cm^2^ at −0.6 eV	
		Fe	55.91 μF/cm^2^ at −0.35 eV	
		Ni	59.74 μF/cm^2^ at 0.29 eV	
		Co	31.40 μF/cm^2^ near 0 V	
		Ti	41.36 μF/cm^2^ near 0 V	
		vanadium	33.54 μF/cm^2^ near 0 V	
		Mn	36.94 μF/cm^2^ near 0 V	
		Cu	52.73 μF/cm^2^ at −0.47 eV	
		Zn	50.87 μF/cm^2^ at −0.11 eV	
transition metal dichalcogenides (TMD)	monolayer MoS_2_^84^	S substitution by N	203.047 μF/cm^2^ at Fermi energy	*ab initio* DFT
		S substitution by F	139 μF/cm^2^ at Fermi energy	
		S substitution by Cl	252 μF/cm^2^ at Fermi energy	
		S substitution by As	189.672 μF/cm^2^ at Fermi energy	
		S substitution by Sb	188.955 μF/cm^2^ at Fermi energy	
		S substitution by Se	0.595 μF/cm^2^ at Fermi energy	
		Mo substituted by Co	152.794 μF/cm^2^ at Fermi energy	
		Mo substituted by Cu	191.658 μF/cm^2^ at Fermi energy	
		Mo substituted by Ni	202.439 μF/cm^2^ at Fermi energy	
		Mo substituted by vanadium	263.721 μF/cm^2^ at Fermi energy	
		5.5% S vacancy	0.190 μF/cm^2^ at Fermi energy	
		11% S vacancy	2.5 μF/cm^2^ at Fermi energy	
		16.5% S vacancy	33.665 μF/cm^2^ at Fermi energy	
		11.5% Mo vacancy	209.733 μF/cm^2^ at Fermi energy	
	three-layered 1T-MoS_2_^115^	pristine*	2063.49 F/g at −0.2 V	*ab initio* DFT HASI, horizontally aligned similar ions; DASI, diagonally aligned similar ions; and VASI, vertically aligned similar ions
		H^+^ ion intercalated*	2419.04 F/g at 0.005 V	
		Li^+^ ion intercalated*	2342.85 F/g at 0.005 V	
		Na^+^ ion intercalated*	2787.30 F/g at −0.002 V	
		K^+^ ion intercalated*	2736.5 F/g at −0.002 V	
	three-layered 2H-MoS_2_^115^	pristine*	772.09 F/g at −0.5 V	
		H^+^ ion intercalated*	474.42 F/g at −0.008 V	
		K^+^ ion intercalated*	3302.32 F/g at 0.3 V	
		Li^+^ ion intercalated*	3004.65 F/g at 0.3 V	
		Na^+^ ion intercalated*	3246.51 F/g at 0.3 V	
		LiNa intercalated (HASI)	3163 F/g at 0.3 V	
		LiNa intercalated (DASI)	3111 F/g at 0.3 V	
		LiNa intercalated (VASI)	3143 F/g at 0.3 V	
2D heterostructure	MoS_2_/graphene^83^	pristine	16.36 μF/cm^2^ at −0.2 V	*ab initio* DFT
		carbon vacancy*	74.4 μF/cm^2^ at −0.32 V	
		Mo vacancy*	258.13 μF/cm^2^ at 0.08 V	
		S vacancy*	273.98 μF/cm^2^ at 0.11 V	
		Sc	73.12 μF/cm^2^ at −0.2 V	
		Sc@S vacancy	166.51 μF/cm^2^ at −0.06 V	
		Ti	75.21 μF/cm^2^ at −0.2 V	
		V	48.26 μF/cm^2^ at −0.2 V	
		Cr	23.44 μF/cm^2^ at −0.2 V	
		Mn	77.56 μF/cm^2^ at −0.2 V	
		Fe	206.30 μF/cm^2^ at −0.2 V	
		Fe@Mo vacancy	309.15 μF/cm^2^ at 0.11 V	
		Fe@S vacancy	144.95 μF/cm^2^ at 0.16 V	
		Co	45.97 μF/cm^2^ at −0.2 V	
		Co@Mo vacancy	304.02 μF/cm^2^ at −0.47 V	
		Co@S vacancy	207.94 μF/cm^2^ at −0.18 V	
		Ni	16.19 μF/cm^2^ at −0.2 V	
		Ni@S vacancy	283.11 μF/cm^2^ at 0.37 V	
MXene	Nb_2_C^94^	pristine	324.1 μF/cm^2^ at 0.5 V	*ab initio* DFT
	Ti_2_C^94^	pristine	246.2 μF/cm^2^ at 0.5 V	
	V_2_C^96^	pristine	3465.51 μF/cm^2^ at −0.5 V	
	Mo_2_C^96^	pristine	3243.99 μF/cm^2^ at −0.5 V	
	Ti_3_C_2_^99^	pristine	398.19 μF/cm^2^ at −0.072 V	*ab initio* DFT
		H terminated	212.10 μF/cm^2^ at −0.072 V	
		O terminated	158.87 μF/cm^2^ at −0.072 V	
		F terminated	244.27 μF/cm^2^ at −0.072 V	
		OH terminated	296.28 μF/cm^2^ at −0.072 V	
		Li adsorbed	291.23 μF/cm^2^ at −0.072 V	
		Na adsorbed	359.19 μF/cm^2^ at −0.072 V	
		K adsorbed	289.45 μF/cm^2^ at −0.072 V	
		Ca adsorbed	342.65 μF/cm^2^ at −0.072 V	
		Mg adsorbed	364.69 μF/cm^2^ at −0.072 V	
		Al adsorbed	398.193 μF/cm^2^ at −0.072 V	
		Li adsorbed on F terminated	228.833 μF/cm^2^ at −0.408 V	
		Na adsorbed on F terminated	227.076 μF/cm^2^ at −0.432 V	
		K adsorbed on F terminated	222.581 μF/cm^2^ at 0.048 V	
		Ca adsorbed on F terminated	242.453 μF/cm^2^ at −0.408 V	
		Mg adsorbed on F terminated	224.492 μF/cm^2^ at −0.432 V	
		Al adsorbed on F terminated	488.153 μF/cm^2^ at 0.12 V	
	Ti_2_C^99^	pristine	272.37 μF/cm^2^ at 0.048 V	
		H terminated	135.36 μF/cm^2^ at 0.048 V	
		O terminated	48.22 μF/cm^2^ at 0.048 V	
		F terminated	184.98 μF/cm^2^ at 0.048 V	
		OH terminated	157.79 μF/cm^2^ at 0.048 V	
		Li adsorbed	187.85 μF/cm^2^ at 0.048 V	
		Na adsorbed	152.41 μF/cm^2^ at 0.048 V	
		K adsorbed	219.72 μF/cm^2^ at 0.048 V	
		Ca adsorbed	159.61 μF/cm^2^ at 0.048 V	
		Mg adsorbed	132.96 μF/cm^2^ at 0.048 V	
		Al adsorbed	444.192 μF/cm^2^ at 0.312 V	
		Li adsorbed on F terminated	259.490 μF/cm^2^ at −0.24 V	
		Na adsorbed on F terminated	247.689 μF/cm^2^ at 0.048 V	
		K adsorbed on F terminated	231.497 μF/cm^2^ at 0.048 V	
		Ca adsorbed on F terminated	220.885 μF/cm^2^ at 0.048 V	
		Mg adsorbed on F terminated	226.881 μF/cm^2^ at 0.048 V	
		Al adsorbed on F terminated	252.554 μF/cm^2^ at 0.072 V	
δ-6 borophene^111^		1 layer in aqueous electrolyte	203.09 μF/cm^2^ at −0.6 V	DFT
		4 layers in aqueous electrolyte	600.36 μF/cm^2^ at −0.15 V	
		1 layer in ionic liquid electrolyte	209.24 μF/cm^2^ at −1 V	
		4 layers in ionic liquid electrolyte	663.27 μF/cm^2^ at −1 V	
silicene^107^		pristine	363.66 F/g at −0.5 V	DFT
		monovacancy	711.20 F/g at −0.25 V	
		trivacancy	673.41 F/g at −0.5 V	
		six vacancy	2004.54 F/g at 0.50 V	
germanene^108^		pristine	3.51 μF/cm^2^ at 0 V (minimum)	DFT
		N doped	45.32 μF/cm^2^ at 0.01 V	
		Ti doped	91.47 μF/cm^2^ at 0.20 V	
		Cr doped	40.96 μF/cm^2^ at −0.20 V	
		Mn doped	39.36 μF/cm^2^ at −0.03 V	
		Co doped	59.70 μF/cm^2^ at 0.32 V	
		B/Co doped	146.99 μF/cm^2^ at −0.54 V	
		Co doped + vacancy	123.33 μF/cm^2^ at −0.42 V	
		B/Co doped + vacancy	116.46 μF/cm^2^ at −0.55 V	

a An asterisk represents the data estimated either from the plot using WebPlotDigitizer software authored by Ankit Rohatgi or available data from the reference.

### 11.2 Challenges

On the basis of this discussion, the key challenges that we foresee in this emerging area are outlined as follows:

- The structural stability of doped and defected electrode material systems is quite challenging.^54^ Basically, there is a trade-off relation between a stable system and the best charge storage performance, as evidenced by the theoretical calculation.^109^ For instance, TMN_3_–Si is the most stable system, but ScN_2_–Si showed the highest *C*_Q_ of 224.88 μF/cm^2^.
- For the doped/functionalized graphene, as an example, the total contribution comes from double-layer capacitance, dopant/functionalized-group-assisted pseudocapacitance, and quantum capacitance. It is unclear from the available reports on the theoretical calculation whether the pseudocapacitance part is considered or not. Moreover, space charge capacitance should be added in the calculation, while one is considering few-layer or multi-layer graphene.^123^
- Adding pseudocapacitive materials into the EDLC materials can enhance the specific capacitance dramatically, and one needs to consider the mechanical properties of the composite because pseudocapacitive materials are mostly brittle.
- It is highly desirable to use dopants with a smaller atomic size and shorter bond lengths. Dopants with a larger atomic size may have other effects. For example, the larger bond length of C–P than C–C leads to the lattice expansion of the original graphene symmetry, and the structure may fail to retain its symmetry.^54^ Larger dopants, e.g., Al^3+^ monovacancy on graphene, is clearly not favorable because it has the higher formation energy.^54^
- Hybrid electrode materials have a synergy effect on the total charge storage performance. However, one needs to keep in mind that many components in the heterostructure have more complexity in theoretical computation because it consists of more constituent material along with the presence of the defect and curvature, and sometimes, it has been observed that a heterostructure with fewer components has superior performance over multicomponent heterostructures.

Therefore, an *in-depth* understanding and further insights into the quantum capacitance of electrode materials are increasingly necessary to advance clean energy storage technology, and this trend will continue in the future.

### 11.3 Outlook and Perspective

Certainly, discussing potential future perspectives in research methodologies, both experimental and modeling, in the context of quantum capacitance of 2D-material-based supercapacitor electrodes is essential for keeping pace with the evolving landscape of the field. The following are some key areas to consider:

- Advanced microscopy and microanalysis: The development of *in situ* and *operando* experimental techniques is beneficial to directly observe the quantum capacitance behavior of 2D materials with deeper insights into the underlying mechanisms. Similarly, nanoscale probing techniques, such as atomic force microscopy (AFM) and scanning tunneling microscopy (STM), can be further refined for better control and optimization to study the quantum capacitance properties at the nanoscale. Furthermore, these studies are also essential to probe the reaction environments and the changes within the electrodes.
- Exploration of novel 2D materials: We can explore and synthesize new 2D materials, such as MXene, borophenes, phosphorene, germanene, etc., with tailored electronic properties for superior quantum capacitance characteristics, leading to enhanced supercapacitor electrodes.
- Quantum mechanical modeling: Advancements in theoretical computational techniques, particularly quantum mechanical modeling (e.g., DFT, tight binding, etc.), can be harnessed to predict the quantum capacitance of 2D materials accurately. This can guide experimental efforts and reduce the need for extensive trial-and-error experiments. Moreover, machine learning and artificial intelligence can also play a leading role in modeling quantum capacitance, allowing for the rapid screening of materials and configurations.
- Multiscale modeling: Integrating multiscale modeling approaches that bridge quantum mechanical calculations with meso- and macroscopic simulations (e.g., electronic structure, interfacial charge transfer, defect density, and surface work function) can provide a comprehensive understanding of quantum capacitance effects in real supercapacitor devices.
- Materials engineering: Future research may focus on engineering the band structures of 2D materials through doping, strain engineering, defect engineering, or heterostructure design to tailor their quantum capacitance for specific applications.
- Device integration: Integration of 2D-material-based supercapacitor electrodes into practical devices from small flex devices for personal use to large-scale commercial energy storage, such as wearable electronics or energy storage systems used for hybrid electric vehicles, will be crucial. This involves addressing scalability, stability, and compatibility issues.
- Energy storage system optimization and measurement guidelines: Beyond materials, future research should focus on the holistic optimization of supercapacitor systems, including electrolytes and device architectures, to maximize the benefits of high-quantum capacitance materials. Moreover, one has to pay attention to the electrochemical measurements at current density, potential, and electrolyte concentrations. For example, reporting the specific capacitance of the device and/or electrode equal or above a 20 mV/s scan rate or a 2 A/g current density is more useful. The information on self-discharge, leakage current, and cell voltage considering the potential drop is other important parameters that can be taken into account.
- Standardization and benchmarking: Developing standards and benchmarking protocols for measuring the quantum capacitance values will be important for ensuring consistency and reliability in the field.

Incorporating these future perspectives into research methodologies, both experimental and modeling, will contribute to the continued advancement of 2D-material-based supercapacitor electrodes and their role in the evolving landscape of energy storage technology.
